# Clinical and radiomics parameter prognostication in metastatic uveal melanoma patients treated with hepatic arterial infusion chemotherapy

**DOI:** 10.1093/oncolo/oyaf385

**Published:** 2025-12-22

**Authors:** Tanja Gromke, Juliane Durand, Tamara T Mueller, Felix Neumaier, Sven T Liffers, Heike Richly, Matthias Grubert, Johannes Haubold, Jens Theysohn, Halime Kalkavan, Nikolaos E Bechrakis, Martin Schuler, Rickmer Braren, Benedikt M Schaarschmidt, Jens T Siveke

**Affiliations:** West German Cancer Center, Department of Medical Oncology, University Hospital Essen, University of Duisburg-Essen, Essen 45122, Germany; West German Cancer Center, Bridge Institute of Experimental Tumor Therapy (BIT) and Division of Solid Tumor Translational Oncology (DKTK), University Hospital Essen, University of Duisburg-Essen, Essen 45122, Germany; Institute for Artificial Intelligence and Informatics in Medicine, Technical University of Munich, Munich 81675, Germany; Institute of Radiochemistry and Experimental Molecular Imaging, Faculty of Medicine and University Hospital Cologne, University of Cologne, Cologne 50937, Germany; West German Cancer Center, Bridge Institute of Experimental Tumor Therapy (BIT) and Division of Solid Tumor Translational Oncology (DKTK), University Hospital Essen, University of Duisburg-Essen, Essen 45122, Germany; German Cancer Consortium (DKTK), a Partnership between German Cancer Research Center (DKFZ) and University Hospital Essen, University of Duisburg-Essen, Essen 45122, Germany; West German Cancer Center, Department of Medical Oncology, University Hospital Essen, University of Duisburg-Essen, Essen 45122, Germany; West German Cancer Center, Department of Medical Oncology, University Hospital Essen, University of Duisburg-Essen, Essen 45122, Germany; Institute of Diagnostic and Interventional Radiology and Neuroradiology, University Hospital Essen, University of Duisburg-Essen, Essen 45122, Germany; Institute of Diagnostic Radiology, Interventional Radiology and Nuclear Medicine, Berufsgenossenschaftliches Universitätsklinikum Bergmannsheil GmbH, Ruhr University Bochum, Bochum 44789, Germany; West German Cancer Center, Department of Medical Oncology, University Hospital Essen, University of Duisburg-Essen, Essen 45122, Germany; National Center for Tumor Diseases (NCT) West, University Hospital Essen, University of Duisburg-Essen, Essen 45122, Germany; Department of Ophthalmology, University Hospital Essen, University of Duisburg-Essen, Essen 45122, Germany; West German Cancer Center, Department of Medical Oncology, University Hospital Essen, University of Duisburg-Essen, Essen 45122, Germany; German Cancer Consortium (DKTK), a Partnership between German Cancer Research Center (DKFZ) and University Hospital Essen, University of Duisburg-Essen, Essen 45122, Germany; National Center for Tumor Diseases (NCT) West, University Hospital Essen, University of Duisburg-Essen, Essen 45122, Germany; Department of Diagnostic and Interventional Radiology, School of Medicine, Technical University of Munich, Munich 81675, Germany; German Cancer Consortium (DKTK), a Partnership between German Cancer Research Center (DKFZ) and School of Medicine, Technical University of Munich, Munich 81675, Germany; German Cancer Consortium (DKTK), a Partnership between German Cancer Research Center (DKFZ) and University Hospital Essen, University of Duisburg-Essen, Essen 45122, Germany; Institute of Diagnostic and Interventional Radiology and Neuroradiology, University Hospital Essen, University of Duisburg-Essen, Essen 45122, Germany; West German Cancer Center, Department of Medical Oncology, University Hospital Essen, University of Duisburg-Essen, Essen 45122, Germany; West German Cancer Center, Bridge Institute of Experimental Tumor Therapy (BIT) and Division of Solid Tumor Translational Oncology (DKTK), University Hospital Essen, University of Duisburg-Essen, Essen 45122, Germany; German Cancer Consortium (DKTK), a Partnership between German Cancer Research Center (DKFZ) and University Hospital Essen, University of Duisburg-Essen, Essen 45122, Germany; National Center for Tumor Diseases (NCT) West, University Hospital Essen, University of Duisburg-Essen, Essen 45122, Germany

**Keywords:** metastatic uveal melanoma, hepatic arterial infusion chemotherapy, multi-variate analysis, independent prognostic factors, machine learning, radiomics

## Abstract

**Introduction:**

Metastatic uveal melanoma (MUM) has a poor prognosis, but hepatic arterial infusion chemotherapy (HAIC) may improve outcomes in patients with hepatic metastases. To identify reliable prognostic factors for patient stratification and treatment allocation, we analyzed the clinical and imaging data from a large single-center cohort using machine learning (ML) models.

**Methods:**

Pre– and post–first treatment clinical data of 235 patients with MUM treated with HAIC between 2009 and 2019 were retrospectively analyzed using Cox regression to identify prognostic factors for overall survival (OS) and time to change treatment strategy (TTCS). Furthermore, ML models were trained on clinical and computed tomography (CT) data for endpoint prediction.

**Results:**

Pre-treatment multi-variate analysis identified elevated lactate dehydrogenase (LDH) (OS: 6.5 vs. 16.4 months, hazard ratio [HR]) = 1.87, *P* = 0.006) and gamma-glutamyl transpeptidase (GGT) (OS: 7.6 vs. 16.4 months, HR = 1.67, *P* = 0.012) as prognostic factors for inferior OS. Decreased albumin (TTCS: 1.3 vs. 6.1 months, HR = 6.26, *P* < 0.001) and elevated LDH (TTCS: 2.9 vs. 7.6 months, HR = 1.72, *P* = 0.011) and alanine aminotransferase (ALT) (TTCS: 3.7 vs. 6.4 months, HR = 1.65, *P* = 0.004) predicted shorter TTCS. Scoring enhanced the power of the prognosticators for OS and TTCS. Post–first treatment multi-variate analysis emphasized the importance of inflammation management and liver protection. ML models incorporating radiomics features from baseline CT imaging were not superior to models based on pre-treatment clinical data alone.

**Conclusion:**

We identified independent but synergistic prognostic factors for outcome stratification to guide treatment decisions and optimize patient management. ML-based radiomics features did not significantly enhance prognostic performance.

Implications for Practice For patients with metastatic uveal melanoma treated with hepatic arterial infusion chemotherapy, routine laboratory parameters are accessible and reliable prognostic tools—particularly in the context of a scoring system. Elevated lactate dehydrogenase (LDH) and gamma-glutamyl transpeptidase (GGT) identify patients with reduced overall survival, while low albumin and elevated LDH and alanine aminotransferase (ALT) predict early treatment termination. These markers can guide risk-adapted treatment planning. Supportive management focusing on liver protection and inflammation control can improve treatment outcomes. Although machine learning–based radiomics did not enhance prognostic accuracy in this study, future approaches using segmentation of metastatic lesions rather than the whole liver may improve predictive performance.

## Introduction

Uveal melanoma (UM), thus rare, is the most common ocular malignancy in the Western world. Fifty percent of patients develop metastatic uveal melanoma (MUM) within 5-10 years, particularly in the liver.^1–7^ Systemic chemotherapy and immune checkpoint blockade have poor outcomes, and tebentafusp treatments are limited by HLA type dependency. Few patients with hepatic metastases benefit from surgery.^8–11^ Liver-directed hepatic arterial infusion chemotherapy (HAIC) significantly improves progression-free survival (PFS). However, prolonged overall survival (OS) is less frequently observed.^12–15^ Reliable prognostic factors are needed to tailor this strategy to specific subgroups, as few have been validated in MUM, whereas they are well established for primary UM.^16–22^ Although manual analysis of routine imaging in patients treated with HAIC provides additional prognostic information, imaging patterns are heterogeneous, and image-based stratification has not been validated. Machine learning (ML)-based radiomics can integrate the non-intuitive imaging data content, but the requirement for large data sets has limited its application in rare cancers. This study evaluated routine clinical data as potential prognostic markers for stratifying patients with hepatic metastases from metastasized UM undergoing HAIC. Additionally, we conducted a proof-of-concept analysis to assess the predictive performance of different ML models trained on clinical and computed tomography (CT) data.

## Methods

### Study design and patient population

This retrospective single-center database analysis of 235 consecutive patients with MUM treated with HAIC at the West German Cancer Center Essen between 2009 and 2019, using data from the local electronic health record and picture archiving and communication system, was approved by the local Ethics Committee (IRB#: 20-9484-BO).

### Hepatic arterial infusion chemotherapy

HAIC of the liver follows a standardized procedure.^23^ Briefly, access was usually 1 femoral artery, with a microcatheter placed in the proper hepatic artery or separately in its branches to ensure chemoperfusion of the entire liver, which is important for targeting potential micro metastases. A fixed dose of 40 mg melphalan was administered in each session. Long-term therapy was repeated every 7 weeks until progression or severe side effects occurred. Patients requiring additional chemoembolization or dose increases were considered to have reached the endpoints.

### Clinical variables

The study analyzed demographics, medical history, performance status, laboratory parameters, and CT scans as detailed in the supplements. Laboratory parameters were dichotomized at normal range limits, medians (neutrophil-to-lymphocyte ratio [NLR] and thrombocyte-to-lymphocyte ratio [TLR]), or clinically relevant thresholds (aspartate aminotransferase [AST], alanine aminotransferase [ALT], gamma-glutamyl transpeptidase [GGT], alkaline phosphatase [AP], and lactate dehydrogenase [LDH]). Continuous demographic or medical history parameters were dichotomized by the median, and categorical variables according to their status.

### Statistical analysis

Median OS and time to change treatment strategy (TTCS) were estimated using the Kaplan–Meier method and compared between groups using the log-rank test. Prognostic factors for OS and TTCS were identified by uni- and multi-variate Cox regression analyses (significance: *P* < 0.05). Calculations used SPSS Statistics V29.0 (IBM, Armonk, United States) without sample size calculations or adjustments for multiple testing due to the exploratory design of the study.

### Imaging data acquisition

Contrast-enhanced liver CT scans (arterial and portal venous phase) were routinely obtained immediately before (pre-perfusion data) and in the mean 7 weeks after the first HAIC (post-perfusion data). Axial CT images with a 5 mm slice thickness were used for radiomics analysis.

### ML-based predictions

Supplementary information details the data segmentation, image post-processing, radiomics feature extraction, and ML modeling. Briefly, following whole liver segmentation with an in-house U-Net algorithm, 2 alternative feature extraction pipelines for CT data were evaluated. The first utilized radiomics features from pre- and post-perfusion liver segmentations using PyRadiomics (version 3.0.1),^24^ resulting in 107 features. The second utilized deep learning features from the U-Net bottleneck used for liver segmentation (bottleneck features), resulting in 4.9 million features per subject. Those features were normalized, and the feature vector was reduced to 100 via a Principle Component Analysis using Python′s sklearn.^25^

To predict binary survival (above versus below median OS) or TTCS (0-3, 3-6, 6-12, or >12 months) from the pre-, post- or pre- and post-perfusion data, a random forest classifier or a multilayer perceptron (MLP) were trained on clinical data, imaging features (PyRadiomics or bottleneck features), clinical data plus imaging features, or clinical data plus random values. Clinical data included prognostic laboratory parameters from the present or previous studies.^13^^,^^18–20^

The classification performance was quantified using sensitivity, specificity, precision, and accuracy. For TTCS prediction, the accuracy for each class was calculated, while the “global” performance was quantified by summing the individual true positive (TP), true negative (TN), false positive (FP), and false negative (FN) values for each fold across all 4 classes to determine micro-averaged performance metrics and the overall accuracy. Differences were tested using 1-way ANOVA with Fisher’s least significant difference test.

## Results

### Demographic and clinical characteristics

A total of 235 patients with MUM, with a median age of 60 years at UM diagnosis and 64 years at the first HAIC, were included. Table 1 presents the key demographic and clinical characteristics.

**Table 1.** Summary of demographic and clinical characteristics.

Characteristic	Number of patients (%) or median value [95% CI]
**Total number of patients**	235 (100)
**Sex**	
** Male**	113 (48)
** Female**	122 (52)
**Age at diagnosis of UM [years]**	60 [57-62]
**Age at diagnosis of MUM [years]**	63 [61-65]
**OS after diagnosis of UM [months]**	49.6 [45.0-59.2]
**OS after diagnosis of MUM [months]**	16.0 [13.8-17.5]
**Age at first HAIC [years]**	64 [62-66]
**Treatment of primary tumor:**	
** Enucleation**	67 (29)
** Ruthenium brachytherapy**	116 (49)
** Other**	52 (22)
**Prior treatments for MUM:**	
** None**	159 (68)
** ≥ 1 prior systemic treatment**	76 (32)
** Chemotherapy**	16 (7)
** Immunotherapy**	33 (14)
** Clinical trial**	36 (15)
** Liver resection**	20 (8)
** Other liver-directed treatments**	5 (2)
**ECOG status:**	
** 0**	155 (66)
** 1**	69 (29)
** ≥2**	11 (5)

Abbreviations: ECOG, Eastern Cooperative Oncology Group; HAIC, hepatic arterial infusion chemotherapy; MUM, metastatic uveal melanoma; OS, overall survival; UM, uveal melanoma.

**Figure 1.**
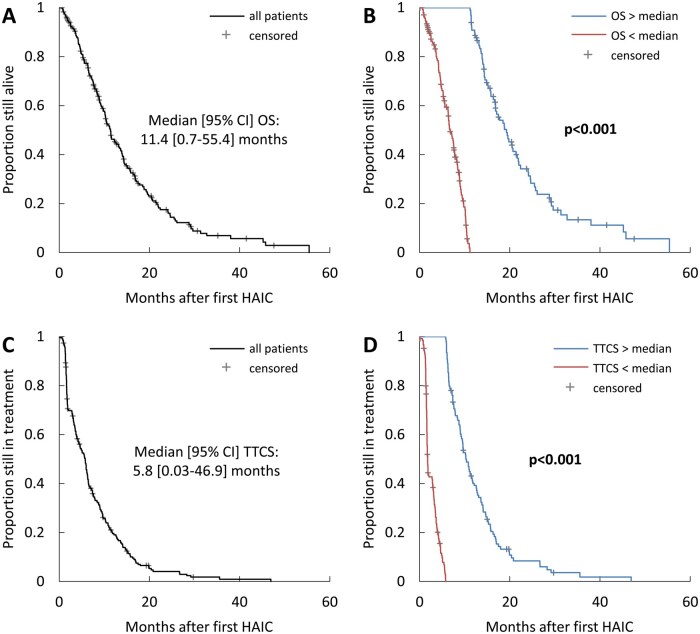
Kaplan–Meier time-to-event curves for overall survival (OS) and time to change treatment strategy (TTCS). Shown are Kaplan–Meier curves for (A) OS in the entire cohort, (B) OS in patients stratified based on the median value in the entire cohort, (C) TTCS in the entire cohort, and (D) TTCS in patients stratified based on the median value in the entire cohort. The *P-*values given in B and D were determined by comparison of the respective groups using the log-rank test.

The median OS after the first HAIC was 11.4 [0.7-55.4] months. Patients above this median lived 19.3 [16.6-22.0] months, compared to 6.8 [5.9-7.7] months for those below (Figure 1A and B). The median TTCS was 5.8 [0.03-46.9] months, with patients above this median at 10.3 [9.1-11.5] months and below at 5.8 [4.9-6.6] months (Figure 1C and D).

### OS analysis of pre-treatment clinical variables

Multi-variate analysis of pre-treatment variables with significant univariate interactions (Table S1) identified LDH and GGT as independent prognostic factors influencing OS (Table 2). Elevated LDH had a stronger impact, with a hazard ratio (HR) of 1.87 [1.19-2.94] (*P* = 0.006) and a median OS of 6.6 [5.4-7.7] months versus 16.4 [13.7-19.1] months for normal LDH. Elevated GGT showed an HR of 1.67 [1.12-2.47] (*P* = 0.012) and a median OS of 7.6 [5.9-9.4] months versus 16.4 [13.9-18.9] months for normal GGT.

**Table 2.** Results of multi-variate analysis of overall survival (OS).

Subgroups		*n*	Median OS [95% CI]	HR [95% CI]	*P*-value
**Hb**	Normal	189	11.58 [10.00-13.16]	1	0.068
	<12 g/dL	44	6.61 [5.41-7.82]	1.49 [0.97-2.28]	
**NLR**	≤Median (5)	182	12.40 [10.52-14.28]	1	0.140
	>Median (5)	46	5.23 [3.31-7.15]	1.44 [0.89-2.32]	
**TLR**	≤Median (175)	107	14.87 [11.68-18.06]	1	0.067
	>Median (175)	120	8.49 [6.50-10.47]	1.40 [0.98-2.01]	
**AST**	≤35 U/L	109	15.13 [12.67-17.59]	1	0.839
	>35 U/L	123	8.22 [7.03-9.42]	0.96 [0.61-1.49]	
**ALT**	≤35 U/L	121	14.24 [12.71-15.78]	1	0.129
	>35 U/L	111	8.45 [6.88-10.03]	1.35 [0.92-2.00]	
**GGT**	≤70 U/L	104	16.38 [13.85-18.91]	1	**0.012**
	>70 U/L	104	7.63 [5.87-9.39]	1.67 [1.12-2.47]	
**AP**	≤100 U/L	89	16.38 [13.79-18.98]	1	0.619
	>100 U/L	67	7.63 [6.43-8.84]	0.90 [0.59-1.37]	
**LDH**	≤400 U/L	123	16.38 [13.66-19.10]	1	**0.006**
	>400 U/L	87	6.55 [5.44-7.65]	1.87 [1.19-2.94]	
**CRP**	Normal	113	15.23 [12.68-17.78]	1	0.279
	≥0.5 mg/dL	110	8.45 [7.15-9.76]	1.23 [0.85-1.79]	
**Karnofsky Performance Status**	90%-100%	148	11.58 [9.44-13.71]	1	
	70%-80%	70	11.22.[9.15-13.28]	1.06 [0.74-1.53]	0.742
	≤60%	8	3.72 [3.47-4.00]	1.12 [0.48-2.58]	0.798
**Prior treatments in metastatic UM**	0	159	13.16 [11.21-15.11]	1	
	1	62	10.16 [8.68-11.65]	1.04 [0.73-1.49]	0.826
	≥2	14	8.22 [0.00-16.89]	1.79 [0.85-3.77]	0.126
**Growth pattern**	Nodular	113	13.36 [10.95-15.76]	1	
	Infiltrative	28	8.22 [2.61-13.84]	1.03 [0.62-1.72]	0.910
	Mixed	82	10.16 [8.93-11.40]	1.17 [0.80-1.70]	0.429
**Hepatic metastases in size**	<5 cm	143	12.27 [9.61-14.93]	1	0.585
	≥5 cm	74	10.10 [8.04-12.16]	1.11 [0.77-1.60]	
**Necrosis**	No	210	11.45 [9.38-13.52]	1	0.729
	Yes	13	9.11 [6.94-11.29]	0.88 [0.43-1.80]	
**Portal vein involvement**	No	204	12.27 [10.58-13.96]	1	0.332
	Yes	7	3.95 [3.61-4.29]	1.67 [0.59-4.67]	
**Other vessel involvement**	No	199	12.27 [10.56-13.98]	1	0.620
	Yes	12	5.33 [3.43-7.23]	1.20 [0.58-2.51]	
**Ascites**	No	189	13.36 [11.42-15.29]	1	0.201
	Yes	21	6.45 [4.03-8.87]	1.48 [0.81-2.71]	

Abbreviations: ALT, alanine aminotransferase; AP, alkaline phosphatase; AST, aspartate aminotransferase; CRP, C-reactive protein; GGT, gammaglutamyl transpeptidase; Hb: hemoglobin; HR, hazard ratios; LDH, lactate dehydrogenase; NLR, neutrophil-to-lymphocyte ratio; TLR, thrombocyte-to-lymphocyte ratio; UM, uveal melanoma. Significant values with p<0.05 written in bold.

**Figure 2.**
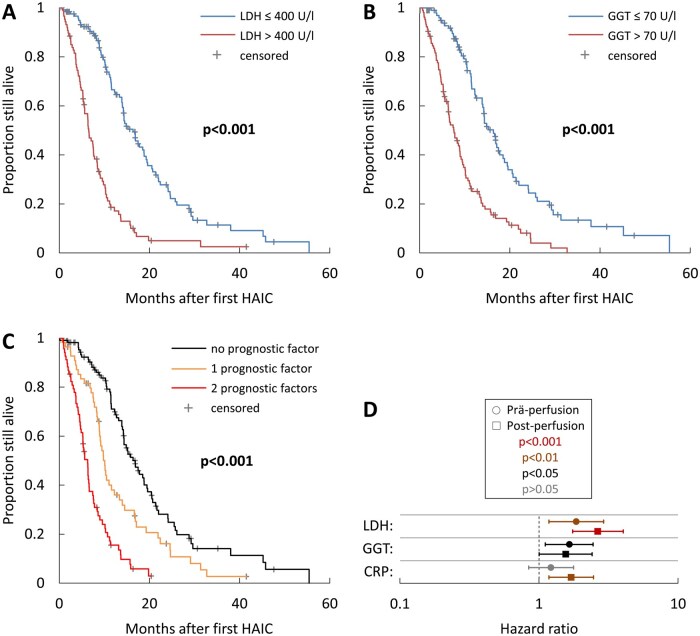
Kaplan–Meier time-to-event curves for overall survival (OS) in relation to independent prognostic variables identified in multi-variate analysis. Shown are Kaplan–Meier curves for OS in patients stratified based on (A) pre-treatment lactate dehydrogenase (LDH) levels, (B) pre-treatment gamma-glutamyl transpeptidase (GGT) levels, and (C) presence of one or more of the unfavorable prognostic factors identified in A and B. The indicated *P*-values were determined by comparison of the different groups using the log-rank test. Forest plot (D) showing the hazard ratios (HR) for LDH, GGT and CRP pre- and post-first perfusion. The confidence interval bars show the 95% confidence intervals of the HR.

We performed Kaplan–Meier analysis for OS on the 2 independent prognostic variables (Figure 2A and B). After stratification, the median OS for patients was 16.8 [14.0-19.7] months with no, 10.1 [8.9-11.3] months with 1 and 6.3 [5.2-7.4] months with both unfavorable prognostic factors (*P* < 0.001 for all comparisons) (Figure 2C).

### TTCS analysis of pre-treatment clinical variables

Multi-variate analysis of pretreatment variables that significantly interacted with TTCS in univariate analysis (Table S2) identified albumin, LDH, and ALT as independent prognosticators (Table 3). Low albumin had the strongest impact, with an HR of 6.26 [2.40-16.36] (*P* < 0.001) and a median TTCS of 1.4 [1.0-1.7] months versus 6.1 [5.5-6.7] months for normal albumin. Additional negative prognosticators included elevated LDH (median TTCS: 2.9 [1.6-4.2] months; HR 1.72 [1.13-2.61], *P* = 0.011) compared to normal LDH (median TTCS: 7.6 [5.8-9.3] months), and elevated ALT (median TTCS: 3.7 [2.5-4.9] months; HR 1.65 [1.17-2.34], *P* = 0.004) compared to normal ALT (median TTCS: 6.5 [5.3-7.6] months).

**Table 3.** Results of multi-variate analysis of time to change strategy (TTCS).

Subgroups		*n*	Median TTCS [95% CI]	HR [95% CI]	*P*-value
**Hb**	Normal	189	5.99 [5.45-6.53]	1	0.228
	<12 g/dL	44	3.16 [2.23-4.08]	1.27 [0.86-1.89]	
**NLR**	≤Median (5)	182	6.12 [5.50-6.74]	1	0.050
	>Median (5)	46	1.91 [0.79-3.03]	1.50 [1.00-2.25]	
**TLR**	≤Median (175)	107	7.20 [5.37-9.04]	1	0.130
	>Median (175)	120	3.55 [2.65-4.46]	1.28 [0.93-1.76]	
**AST**	≤35 U/L	109	7.20 [5.60-8.80]	1	0.341
	>35 U/L	123	3.55 [2.13-4.98]	0.83 [0.55-1.23]	
**ALT**	≤35 U/L	121	6.45 [5.28-7.62]	1	**0.004**
	>35 U/L	111	3.68 [2.47-4.90]	1.65 [1.17-2.34]	
**GGT**	≤70 U/L	104	7.60 [5.63-9.57]	1	0.263
	>70 U/L	104	3.29 [2.35-4.23]	1.22 [0.86-1.75]	
**AP**	≤100 U/L	89	16.38 [13.79-18.98]	1	0.331
	>100 U/L	67	7.63 [6.43-8.84]	0.83 [0.57-1.21]	
**LDH**	≤400 U/L	123	7.57 [5.83-9.30]	1	**0.011**
	>400 U/L	87	2.86 [1.56-4.17]	1.72 [1.13-2.61]	
**Albumin**	Normal	150	6.09 [5.52-6.66]	1	**<0.001**
	<3.2 g/dL	5	1.35 [1.00-1.70]	6.26 [2.40-16.36]	
**CRP**	Normal	113	7.57 [5.82-9.32]	1	0.114
	≥0.5 mg/dL	110	3.52 [2.86-4.18]	1.30 [0.94-1.80]	
**Hepatic nodule size**	<5 cm	143	6.18 [5.21-7.16]	1	0.328
	≥5 cm	74	3.82 [2.28-5.35]	1.17 [0.85-1.60]	
**Portal vein involvement**	No	204	6.12 [5.64-6.60]	1	0.670
	Yes	7	1.91 [1.49-2.33]	1.20 [0.51-2.83]	
**Ascites**	No	189	6.18 [5.71-6.66]	1	0.185
	Yes	21	2.07 [0.50-3.65]	1.42 [0.85-2.37]	

Abbreviations: ALT, alanine aminotransferase; AP, alkaline phosphatase; AST, aspartate aminotransferase; CRP, C-reactive protein; GGT, gamma-glutamyl transpeptidase; Hb, hemoglobin; HR, hazard ratios; LDH, lactate dehydrogenase; NLR, neutrophil-to-lymphocyte ratio; TLR, thrombocyte-tolymphocyte ratio. Significant values with p<0.05 written in bold.

**Figure 3.**
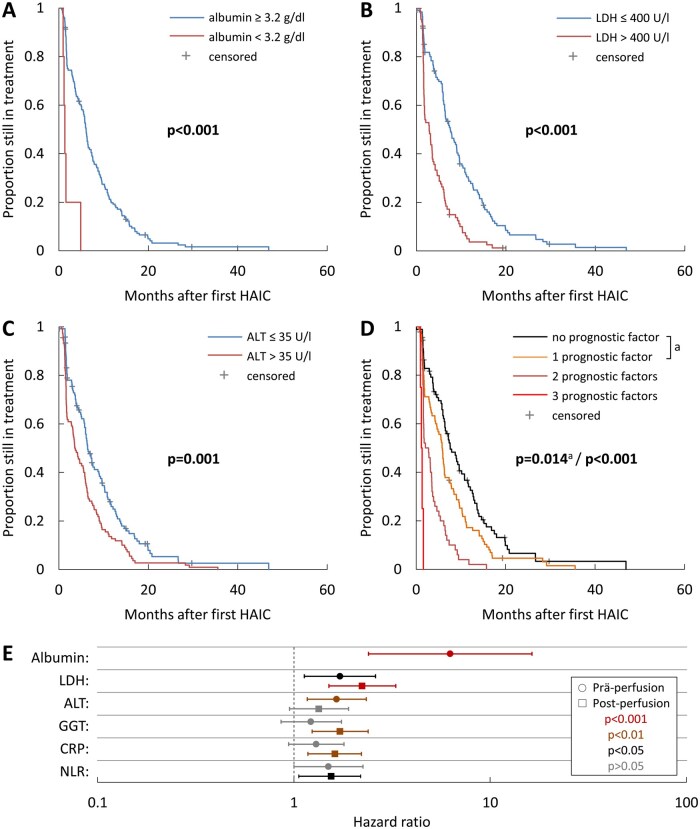
Kaplan–Meier time-to-event curves for time to change treatment strategy (TTCS) in relation to independent prognostic variables identified in multi-variate analysis. Shown are Kaplan–Meier curves for TTCS in patients stratified based on (A) pre-treatment albumin levels, (B) pre-treatment lactate dehydrogenase (LDH) levels, (C) pre-treatment alanine aminotransferase (ALT) levels, and (D) presence of one or more of the unfavorable prognostic factors shown in A-C. The indicated *P*-values were determined by comparison of the different groups using the log-rank test. Forest plot (E) showing the hazard ratios (HR) for albumin, LDH, ALT, gamma-glutamyl transpeptidase (GGT), CRP, and NLR pre- and post-first perfusion. The confidence interval bars show the 95% confidence intervals of the HR. The post-perfusion values for albumin are missing as the value was no longer evaluable after the first perfusion since most patients with this negative predictor had already stopped therapy at this timepoint.

Figure 3A-C shows Kaplan–Meier curves for TTCS in relation to the 3 independent prognostic variables. After stratification (Figure 3D) the median TTCS for patients was 7.6 [5.4-9.9] months with no, 5.9 [5.0-6.8] months with 1, 2.1 [0.7-3.5] months with 2 and 1.2 [0.8-1.6] months with all 3 unfavorable prognostic factors (*P* = 0.014 for no vs. 1 factor, *P* < 0.001 for all others).

### OS analysis of post–first treatment clinical variables

Next, we analyzed the clinical parameters after treatment initiation, taking into account the treatment response patterns. Multi-variate analysis of post–first treatment variables with significant univariate interaction identified LDH, CRP, and GGT as independent prognostic factors influencing OS (Tables S3 and S4). LDH had the strongest impact, with an HR of 2.67 [1.76-4.07] (*P* < 0.001) for elevated LDH (OS: 6.3 [5.2-7.4] months) relative to normal LDH (OS: 16.8 [14.5-19.2] months). The HR for elevated CRP (OS: 8.5 [6.7-10.3] months) relative to normal CRP (OS: 15.8 [13.3-18.4] months) was 1.72 [1.19-2.49] (*P* = 0.004). The HR for elevated GGT (OS: 7.8 [6.5-9.1] months) relative to normal GGT (OS: 16.9 [13.7-20.1] months) was 1.57 [1.01-2.43] (*P* = 0.043). A forest plot summarizes the HRs with confidence intervals and compares them with pre-treatment data (Figure 2D).

We performed Kaplan–Meier analysis for OS with post–first treatment data in relation to the 3 independent prognostic variables (Figure S1A-C, see online supplementary material for a color version of this figure). After stratification (Figure S1D, see online supplementary material for a color version of this figure) the median OS for patients was 17.5 [15.4-19.6] months with no, 13.4 [10.4-16.3] months with 1 and 9.0 [7.6-10.5] months with 2 and 5.6 [4.6-6.5] months with all 3 unfavorable prognostic factors (*P* = 0.153 for no vs. 1, *P* = 0.003 for 2 vs. 3 factors and *P* < 0.001 for all others).

### TTCS analysis of post–first treatment clinical variables

Multi-variate analysis of post–first treatment variables that significantly interacted with TTCS in univariate analysis identified LDH, GGT, CRP, and NLR as independent prognosticators (Tables S5 and S6). Elevated LDH had the strongest impact, with an HR of 2.23 [1.51-3.31] (*P* < 0.001) and a median TTCS of 1.9 [0.9-2.8] months compared to 7.8 [6.1-9.5] months for normal LDH. Additional negative prognosticators included elevated GGT (median TTCS: 3.5 [2.4-4.5] months; HR 1.72 [1.24-2.39], *P* = 0.001) compared to normal GGT (median TTCS: 9.0 [7.4-10.7] months), and elevated CRP (median TTCS: 3.3 [2.7-3.9] months; HR 1.62 [1.18-2.21], *P* = 0.003) compared to normal CRP (median TTCS: 7.8 [6.1-9.5] months) as well as elevated NLR (median TTCS: 3.1 [2.3-4.0] months; HR 1.55 [1.06-2.19], *P* = 0.023) compared to normal NLR (median TTCS: 7.4 [6.2-8.5] months). A forest plot summarizes the HRs with confidence intervals and compares them with pre-treatment data (Figure 3E).

Figure S2A-D (see online supplementary material for a color version of this figure) shows Kaplan-Meier curves for TTCS in relation to the 4 independent prognostic variables. After stratification (Figure S2E, see online supplementary material for a color version of this figure) the median TTCS for patients was 7.8 [5.6-10.0] months with no, 8.4 [5.4-11.4] months with 1, 6.0 [5.1-6.8] months with 2, 2.9 [0.8-4.9] months with 3 and 1.8 [1.6-2.0] months with all 4 unfavorable prognostic factors (*P* = 0.945 for no vs. 1, *P* = 0,003 for 1 vs. 2, *P* = 0,004 for 2 vs. 3, *P* = 0.053 for 3 vs. 4 factors, *P* < 0.001 for all others).

### ML-based predictions

We assessed the performance metrics of ML-based models obtained by fitting a random forest classifier to clinical and/or radiomics features collected before (PRE data), after (POST data), or before and after the first HAIC (PRE+POST data) for OS prediction (Table S7).

**Figure 4.**
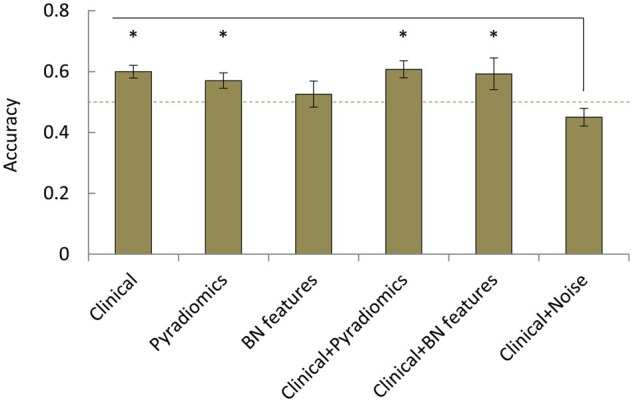
Accuracy of machine learning (ML) models for prediction of overall survival (OS) based on the pre-treatment data. ^*^P< .05 as determined by one-way ANOVA with Fisher’s least significant ­difference test.

Compared to pre-treatment data alone, post-treatment data alone or in combination with pre-treatment data did not improve the performance. Models using either clinical data, radiomics features extracted from whole liver segmentations (PyRadiomics) or the combination of both showed a significantly better accuracy than models based on clinical data and random values (Figure 4). In contrast, radiomics features from the bottleneck layer of the deep learning algorithm (bottleneck features) alone did not perform significantly better than those based on clinical data and random values. Moreover, models based on radiomics features showed a comparable (PyRadiomics) or worse (bottleneck features) accuracy when compared to models based on clinical data, and combining clinical data and radiomics features did not significantly enhance the performance. Although multi-class classifications to predict TTCS showed similar results, their overall performance was particularly poor (overall accuracy < 0.5, Tables S8 and S9). MLPs performed worse or, at best, comparable to random forest classifiers (data not shown).

## Discussion

MUM remains an aggressive disease with limited options despite growing scientific attention. Therefore, assigning the individual to the optimal treatment is crucial. To provide clinicians with practical tools for treatment allocation, we analyzed patients with metastasized UM treated with HAIC to identify clinical prognostic factors and to assess whether ML models enhance prediction. The West German Cancer Center treats one of the largest MUM cohorts in Europe. To our knowledge, this retrospective case series represents the largest single-center analysis of patients with MUM undergoing HAIC to date.

While the median OS after the first HAIC in our cohort was 11.4 months, a substantial proportion of 39% of patients showed a more indolent course of disease (median OS: 19.3 vs. 6.8 months). Two distinct survival patterns have been previously observed in patients with MUM.^4^^,^^5^^,^^26^^,^^27^ Now our findings refine this understanding by identifying the clinical characteristics of these patterns. Our analysis of the pre-treatment clinical characteristics identified elevated LDH, reflecting altered lactate metabolism, and GGT levels, as indicators of liver dysfunction, and independent prognostic factors associated with inferior OS. Both have been previously linked to poor survival in MUM—LDH more often than GGT.^18^^,^^20–22^^,^^28^ The absence of both prognostic factors reliably identified long-term survivors (median OS: 16.8 months), whereas the presence of 1 (median OS: 10.1 months) or both (median OS: 6.3 months) factors was associated with shorter OS. Therefore, both parameters aid in identifying high-risk patients who may benefit from intensified treatment.

As some patients discontinued HAIC for reasons unrelated to disease progression, we analyzed TTCS as a secondary outcome parameter. Again, 2 subgroups emerged: median TTCS <6 months versus >10 months. Most univariate prognostic factors overlapped with those relevant for OS. Interestingly, decreased albumin, indicating liver dysfunction, was uniquely linked to inferior TTCS in multi-variate analysis as the strongest independent prognostic factor (HR = 6.26). This suggests albumin’s utility for selecting patients who can tolerate and benefit from HAIC, although based on only 5 patients. Additional variables independently associated with inferior TTCS in multi-variate analysis included elevated LDH and ALT levels (HR = 1.72 and 1.65). Given that elevated LDH was also an independent predictor of poor survival, the association between LDH and TTCS may be related to disease progression. Similarly, elevated ALT was associated with reduced OS in univariate analysis and has been reported as an independent predictor of poor survival in MUM.^29^ These findings indicate that liver function is also crucial for the TTCS endpoint. Additive scoring of these 3 prognostic factors markedly improves the selection of patients, since median TTCS significantly decreased when comparing patients with no (7.6 months), 1 (5.9 months), 2 (2.1 months), or all 3 (1.2 months) unfavorable prognostic factors. As treatment tolerability and duration are strongly influenced by tumor aggressiveness and the extent of liver metastases, these variables also aid in identifying high-risk patients.

In the HAIC treatment series, post–first treatment analysis identified elevated LDH, CRP, and GGT as independent prognostic factors for inferior OS, and again, additive scoring markedly enhanced the prognostic power. OS decreased with each additional factor when comparing patients after the first treatment with no (17.5 months), 1 (13.4 months), 2 (9.0 months), or all 3 (5.6 months) unfavorable prognostic factors. The same applies to the prognostic factors for TTCS with LDH, GGT, CRP, and NLR, now calculated as significant. TTCS decreased when comparing patients after the first treatment with 1 (8.4 months), 2 (6.0 months), 3 (2.9 months), or all 4 (1.8 months) unfavorable prognostic factors. The pre-treatment prognostic factor albumin lost statistical significance post–first treatment as 3 out of 5 patients with low albumin had already discontinued HAIC due to death. This rapid mortality underscores the prognostic value of albumin. CRP has emerged as a significant post–first treatment factor, emphasizing the impact of inflammatory processes caused by HAIC, such as cell damage in tumor and stromal cells. The NLR, which is also affected by tumor- and therapy-related stress, appears less reliable due to additional factors such as melphalan-induced neutropenia and the potential influence of granulocyte colony-stimulating factor.

While the predictors remained mostly unchanged before and after the first therapy, new factors emerged from antitumor effects, therapy-induced liver stress, and additional side effects, making it difficult to precisely determine their individual contributions. Consequently, pre-treatment data are more reliable for prognostication and treatment assignments. One aspect becomes evident: optimizing therapy requires not only adjusting treatment intensity but also focusing on liver protection and inflammation management.

We evaluated whether ML models have predictive value in MUM, as already demonstrated for various cancers.^30–34^ While models for predicting OS based on clinical and/or radiomics features performed significantly better than models based on clinical data and random values, their overall performance was only modest, and combination of clinical data with radiomics features provided no significant advantage over the use of clinical parameters alone. This could be partly attributable to the sample size, which limits the ability to capture complex interactions between radiomics features and clinical parameters. ML models for prognostic applications require large data sets, which remains challenging in rare diseases. Moreover, in deep learning-based radiomics, the extracted features correlate highly with the input data, requiring particularly large data sets for robust feature subset identification.^35^ This may explain why our bottleneck features showed by far the worst performance. These findings align with the failure of manual CT imaging analysis to identify multi-variate significant prognosticators and reflect the heterogeneity of liver metastases in contrast uptake, arterializations, growth pattern, necrosis, and size from micro metastases to large nodes. Functional imaging techniques that consider tumor metabolism may be more effective, but are only infrequently studied. Furthermore, our radiomics features were extracted from whole liver segmentations, which may have diluted lesion-specific features. Algorithms that segment only the liver metastases may better capture relevant imaging biomarkers, and thus yield superior results.

Our study indicates that adding post-treatment clinical data does not improve OS prediction compared with pre-treatment data alone. Here, the comparatively small ML dataset may be 1 cause, as incorporating the post–first treatment data increases the complexity, especially with the inclusion of 2 time points. Additionally, our random forest classifier outperformed multilayer perceptrons, supporting studies demonstrating the robustness of random forests in small data sets.^36^ This suggests that they may be preferable to more complex neuronal networks in MUM.

Single-center data offer strengths in standardization, robust data quality, and efficient study execution. Furthermore, only very few centers offer HAIC for patients with MUM due to its rarity. Even if their protocols showed mostly minor variations, this would have impaired the homogeneity of the cohort in a multi-center design. The retrospective design with inclusion of the pre–tebentafusp era represents limitations, but enable the analysis of a long-term real-world cohort. This is valuable given the rarity of MUM with the challenges of assembling large prospective cohorts, while data collection is inherently slow despite the high clinical need. With limited HLA status data, we could not address HLA-matched differences in the performance of the identified parameters. To date, there are no published data indicating an influence of HLA status on the outcome after HAIC. As combined or sequential local and systemic treatments become more prevalent, future research will focus on factors determining the best outcomes under such strategies.

Given the exploratory nature of our rare disease study, no formal sample size calculation or adjustment for multiple testing was performed. The reported *P*-values should therefore be interpreted cautiously and as hypothesis-generating. OS as the primary endpoint is influenced by the natural course of disease and systemic therapies. Our secondary endpoint, TTCS, has not yet been validated and combines disease progression and treatment intolerance, reflecting real-world decision-making, where both aspects contribute to therapy discontinuation. These endpoints may not fully capture treatment benefit and should be corroborated in prospective studies using standardized PFS or patient-reported outcome measures.

For the machine-learning proof-of-concept, we dichotomized OS at its median and binned TTCS into broad categories to enable classification. This simplification reduces the temporal resolution and is suboptimal because all patients eventually progress or discontinue therapy. Models trained on time-to-event data and larger cohorts are needed to unlock the potential of ML for patient stratification in MUM.

Taken together, the clinical findings of our study should be regarded as exploratory results that need to be validated in prospective multi-center studies.

## Conclusion

Metastasized UM’s aggressive nature with limited therapeutic options makes it essential to provide clinicians with practical tools for therapy assignment and, at the same time to explore innovative approaches. In this large cohort of patients treated with arterial hepatic infusion chemotherapy elevated pre-treatment LDH and GGT levels independently and synergistically predicted reduced OS, aiding in outcome stratification and high-risk patient identification for potential treatment intensification. Decreased pre-treatment albumin and elevated LDH or ALT levels independently and synergistically predicted early treatment termination. The post–first treatment data underscored the importance of liver protection and inflammation management. Finally, our proof-of-concept evaluation of ML-based radiomics features from whole liver segmentations did not significantly enhance prognostic performance, highlighting the need for revised approaches and possibly larger datasets to identify novel predictive biomarkers.

## Supplementary Material

oyaf385_Supplementary_Data

## Data Availability

The aggregated data generated or analyzed during this study are included in this article and its supplementary material files. The underlying raw data are not publicly available due to their nature of including patient-identifying information. Further enquiries can be directed to the corresponding authors.
