# Supplementary material for: Clinical and radiomics parameter prognostication in metastatic uveal melanoma patients treated with hepatic arterial infusion chemotherapy
Source: Oncologist. 2025 Dec 22;31(1):oyaf385. doi: 10.1093/oncolo/oyaf385 (PMC12790439; doi:10.1093/oncolo/oyaf385)
Supplement: oyaf385_Supplementary_Data [file oyaf385_supplementary_data.docx]

**Online Supplementary Material**

***Clinical and radiomics parameter prognostication in metastatic uveal melanoma patients treated with hepatic arterial infusion chemotherapy***

Tanja Gromke^1^, Juliane Durand^2^, Tamara T Mueller^3^, Felix Neumaier^4^, Sven T Liffers^2,5^, Heike Richly^1^, Matthias Grubert^1^, Johannes Haubold^6^, Jens Theysohn^7^, Halime Kalkavan^1^, Nikolaos E Bechrakis^8,9^, Martin Schuler^1,5,8^, Rickmer Braren^10,11^, Benedikt M Schaarschmidt^5,6^, Jens T Siveke^1,2,5,8^

^1^West German Cancer Center, Department of Medical Oncology, University Hospital Essen, University of Duisburg-Essen, 45122 Essen, Germany

^2^West German Cancer Center, Bridge Institute of Experimental Tumor Therapy (BIT) and Division of Solid Tumor Translational Oncology (DKTK), University Hospital Essen, University of Duisburg-Essen, 45122 Essen, Germany

^3^ Institute for Artificial Intelligence and Informatics in Medicine, Technical University of Munich, 81675 Munich, Germany

^4^Institute of Radiochemistry and Experimental Molecular Imaging, Faculty of Medicine and University Hospital Cologne, University of Cologne, 50937 Cologne, Germany

^5^German Cancer Consortium (DKTK), a partnership between German Cancer Research Center (DKFZ) and University Hospital Essen, University of Duisburg-Essen, 45122 Essen, Germany

^6^ Institute of Diagnostic and Interventional Radiology and Neuroradiology, University Hospital Essen, University of Duisburg-Essen, 45122 Essen, Germany

^7^Institute of Diagnostic Radiology, Interventional Radiology and Nuclear Medicine, Berufsgenossenschaftliches Universitätsklinikum Bergmannsheil GmbH, Ruhr University Bochum, Buerkle-de-la-Camp Platz 1, 44789, Bochum, Germany

^8^National Center for Tumor Diseases (NCT) West, University Hospital Essen, University of Duisburg-Essen, 45122 Essen, Germany

^9^ Department of Ophthalmology, University Hospital Essen, University of Duisburg-Essen, 45122 Essen, Germany

^10^Department of Diagnostic and Interventional Radiology, School of Medicine, Technical University of Munich, 81675 Munich, Germany

^11^German Cancer Consortium (DKTK), a partnership between German Cancer Research Center (DKFZ) and School of Medicine, Technical University of Munich, 81675 Munich, Germany

**Correspondence to:** Jens T. Siveke, West German Cancer Center, Department of Medical Oncology, University Hospital Essen, University of Duisburg-Essen, Hufelandstr. 55, 45122 Essen, Germany. E-mail address: [jens.siveke@uk-essen.de](mailto:jens.siveke@uk-essen.de)

**Table of contents**

1. Data segmentation, image post-processing, radiomics feature extraction, and ML modeling	3

2. Supplemental Tables	5

Supplemental Table 1	5

Supplemental Table 2	7

Supplemental Table 3	9

Supplemental Table 4	11

Supplemental Table 5	12

Supplemental Table 6	14

Supplemental Table 7	15

Supplemental Table 8	15

Supplemental Table 9	16

3. Supplemental Figures	17

Supplemental Figure 1	17

Supplemental Figure 2	18

4. References	19

**1. Data segmentation, image post-processing, radiomics feature extraction, and ML modeling**

Pseudonymized datasets were exported from the hospital picture archiving system and whole liver segmentation was performed using an in-house U-Net segmentation algorithm. For subsequent ML modeling, two independent feature extraction pipelines were evaluated. In the first approach, radiomics features were separately derived from the pre- and post-perfusion liver segmentations using the Python package PyRadiomics (version 3.0.1) [1], yielding a total of 107 features comprising first-order statistics, two- and three-dimensional shape-based features, a gray-level co-occurrence matrix (GLCM), a gray-level run length matrix (GLRLM), a gray-level size zone matrix (GLSZM), a neighboring gray tone difference matrix (NGTDM), and a gray-level dependence matrix (GLDM). In the second approach, deep learning features were extracted from the bottleneck of the U-Net used for liver segmentation, resulting in 4.9 million features per subject. Those features were then normalized by min-max normalization and the feature vector was reduced to 100 features (approximately matching the size of the PyRadiomics feature vector) using a Principle Component Analysis (PCA) and the Python package sklearn [2]. Both, the radiomics features and the PCA features from the U-Net bottleneck, were subsequently used separately as input vectors to the ML models.

To predict binary survival (above versus below median OS) or TTCS (0-3 months, 3-6 months, 6-12 months or >12 months) after the first HAIC from the pre-perfusion, post-perfusion or pre- and post-perfusion data, a random forest classifier was fit to either the respective clinical data only, the respective image features (bottleneck features or PyRadiomics features) only, the respective clinical data plus the image features or the respective clinical data plus noise (random values drawn from a Gaussian distribution). The random forest models were built from 100 individual trees and with a maximum depth of 7 layers, using bootstrap samples. As an alternative machine learning method to the random forest classifier, a multilayer perceptron (MLP) was trained to fit the data. The MLP had four linear layers with LeakyReLU activation functions and was trained with Adam optimizer, a learning rate of 0.001, and a weight decay of 0.01. Clinical data used for the classifications were laboratory parameters (LDH, GGT and AP) identified as independent prognostic factors for OS in the present (see results section) and/or previous studies [3–6]. The LDH parameter was split into three classes (low: LDH ≤ 280 U/l; medium: LDH > 280 U/l but ≤ 1000 U/l; high: LDH > 1000 U/l) and missing values were imputed using the IterativeImputer package in scikit-learn [2]. To reduce the impact of the small data set, a 5-fold cross-validation was used for each evaluation. All analyses were performed using the Python programming language.

Performance of the ML-based classifications was quantified in terms of sensitivity, specificity, precision, and accuracy, which were calculated from the number of true positive (TP), true negative (TN), false positive (FP) and false negative (FN) predictions on the test data set from each fold. For prediction of TTCS, the accuracy for each class was calculated, while the ‘global’ performance was quantified by summing the individual TP, TN, FP, and FN values for each fold across all four classes to calculate the corresponding micro-averaged performance metrics. In addition to the average accuracy thus obtained, the overall accuracy was calculated according to

Overall accuracy = (k * Average Accuracy + 2 - k) / 2	[Eq. 1]

where k is the number of classes. All calculations were performed using Microsoft Excel 2010 (Microsoft, Redmond, USA) and the results are expressed as mean ± standard error of the mean (SEM) across all five folds. Statistical significance of the differences between the performance metrics for the various models was identified by one-way ANOVA with Fisher’s least significant difference test.

**2. Supplemental Tables**

**Supplemental Table 1**: Results of univariate pre-treatment analysis of overall survival (OS).

| **Subgroups** |  | **n** | **Median OS [95% CI]** | **HR [95% CI]** | **p-value** |
| --- | --- | --- | --- | --- | --- |
| Leukocytes | normal  > 9.2 /nl | 178  55 | 11.58 [9.71–13.45]  6.81[4.94–8.68] | 1  1.40 [0.99–1.99] | 0.056 |
| Neutrophils | normal  > 6.2 /nl | 168  60 | 12.24 [10.29–14.18]  6.65 [5.13–8.16] | 1  1.48 [1.06–2.08] | **0.021** |
| Lymphocytes | normal  < 1.0 /nl | 192  36 | 11.55 [9.56–13.54]  8.36 [5.98–10.73] | 1  1.87 [1.22–2.88] | **0.004** |
| Thrombocytes | normal  > 380 /nl | 196  37 | 11.55 [9.82–13.27]  6.65 [5.25–8.04] | 1  1.41 [0.94–2.10] | 0.950 |
| Hb | normal  < 12 g/dl | 189  44 | 11.58 [10.00–13.16]  6.61 [5.41–7.82] | 1  2.08 [1.44–2.99] | **<0.001** |
| NLR | ≤ median (5)  > median (5) | 182  46 | 12.40 [10.52–14.28]  5.23 [3.31–7.15] | 1  2.57 [1.77–3.73] | **<0.001** |
| TLR | ≤ median (175)  > median (175) | 107  120 | 14.87 [11.68–18.06]  8.49 [6.50–10.47] | 1  2.06 [1.51–2.80] | **<0.001** |
| Bilirubin | normal  > 1.2 mg/dl | 215  17 | 11.45 [10.04–12.86]  10.30 [3.34–17.26] | 1  1.05 [0.60–1.85] | 0.863 |
| AST | ≤ 35 U/l  > 35 U/l | 109  123 | 15.13 [12.67–17.59]  8.22 [7.03–9.42] | 1  2.13 [1.57–2.89] | **<0.001** |
| ALT | ≤ 35 U/l  > 35 U/l | 121  111 | 14.24 [12.71–15.78]  8.45 [6.88–10.03] | 1  1.62 [1.20–2.17] | **0.002** |
| GGT | ≤ 70 U/l  > 70 U/l | 104  104 | 16.38 [13.85–18.91]  7.63 [5.87–9.39] | 1  2.57 [1.90–3.49] | **<0.001** |
| AP | ≤ 100 U/l  > 100 U/l | 82  83 | 14.38 [12.48–16.27]  7.43 [5.96–8.91] | 1  1.60 [1.18–2.16] | **<0.001** |
| LDH | ≤ 400 U/l  > 400 U/l | 123  87 | 16.38 [13.66–19.10]  6.55 [5.44–7.65] | 1  3.12 [2.29–4.26] | **<0.001** |
| Total protein | normal  < 6.4 g/dl | 200  31 | 11.38 [9.46–13.30]  10.16 [2.10–18.23] | 1  1.25 [0.78–1.99] | 0.356 |
| Albumin | normal  < 3.2 g/dl | 150  5 | 11.45 [9.37–13.52]  2.14 [0.09–4.19] | 1  1.72 [0.64–4.65] | 0.284 |
| CRP | normal  ≥ 0.5 mg/dl | 113  110 | 15.23 [12.68–17.78]  8.45 [7.15–9.76] | 1  2.19 [1.61–2.96] | **<0.001** |
| S-100 | normal  > 0.105 µg/l | 53  51 | 14.21 [9.49–18.93]  10.30 [7.91–12.68] | 1  1.07 [0.76–1.50] | 0.715 |
| ECOG status | 0  1  ≥2 | 151  65  11 | 11.55 [9.04–14.05]  11.45 [8.61–14.28]  3.72 [2.53–4.91] | 1  1.10 [0.79–1.53]  2.55 [1.29–5.05] | 0.582  **0.007** |
| Karnofsky Performance Status | 90-100%  70-80%  ≤ 60% | 148  70  8 | 11.58 [9.44–13.71]  11.22 [9.15–13.28]  3.72 [3.47–4.00] | 1  1.39 [1.02–1.90]  2.50 [1.16–5.39] | **0.037**  **0.019** |
| Sex | male  female | 113  122 | 11.02 [8.59–13.45]  11.38 [9.03–13.74] | 1  1.01 [0.75–1.36] | 0.927 |
| Affected eye | left  right | 116  117 | 11.45 [8.67–14.23]  10.46 [9.10–11.82] | 1  0.94 [0.70–1.26] | 0.664 |
| Age at FDoP | < 60 years  ≥ 60 years | 115  119 | 10.53 [9.39–11.66]  12.40 [9.56–15.24] | 1  0.95 [0.70–1.27] | 0.708 |
| Age at FDoM | < 63 years  ≥ 63 years | 120  115 | 11.22 [10.10–12.34]  11.45 [8.46–14.44] | 1  0.97 [0.72–1.30] | 0.835 |
| Age at start of HAIC | < 64 years  ≥ 64 years | 122  113 | 11.18 [10.05–12.32]  12.40 [9.03–15.77] | 1  0.92 [0.69–1.24] | 0.583 |
| Time between FDoP & FDoM | < 32 months  ≥ 32 months | 118  116 | 11.38 [9.65–13.11]  11.45 [9.02–13.88] | 1  0.84 [0.63–1.14] | 0.263 |
| Weight | < 75 kg  ≥ 75kg | 88  110 | 11.35 [10.05–12.65]  12.73 [10.30–15.17] | 1  1.13 [0.83–1.53] | 0.448 |
| Metastatic sites | 1  2  3  ≥4 | 96  65  37  37 | 11.45 [9.25–13.61]  11.18 [7.18–15.19]  10.10 [8.71–11.49]  13.88 [11.35–16.42] | 1  1.10 [0.76–1.60]  1.21 [0.80–1.85]  0.89 [0.57–1.38] | 0.628  0.373  0.595 |
| Secondary tumors | 0  ≥1 | 200  35 | 11.18 [10.22–12.15]  15.82 [9.88–21.77] | 1  0.75 [0.48–1.17] | 0.199 |
| Comorbidities | 0  1  2  3  ≥4 | 73  60  56  26  20 | 13.59 [10.48–16.69]  10.43 [7.30–13.56]  10.26 [9.04–11.49]  11.18 [9.24–13.13]  12.40 [8.60–16.20] | 1  1.13 [0.77–1.65]  1.51 [1.01–2.28]  1.22 [0.72–2.05]  1.21 [0.65–2.26] | 0.545  **0.046**  0.456  0.559 |
| Prior treatments in metastatic UM | 0  1  ≥2 | 159  62  14 | 13.16 [11.21–15.11]  10.16 [8.68–11.65]  8.22 [0.00–16.89] | 1  1.20 [0.86–1.68]  2.67 [1.42–4.99] | 0.273  **0.002** |
| Growth pattern | nodular  infiltrative  mixed | 113  28  82 | 13.36 [10.95–15.76]  8.22 [2.61–13.84]  10.16 [8.93–11.40] | 1  1.83 [1.17–2.86]  1.44 [1.04–2.00] | **0.008**  **0.029** |
| Hepatic nodule size | < 5cm  ≥ 5cm | 143  74 | 12.27 [9.61–14.93]  10.10 [8.04–12.16] | 1  1.54 [1.12–2.10] | **0.007** |
| Density | Isodens  hypodens  hyperdens | 7  202  6 | 12.40 [0.00–31.48]  11.35 [9.39–13.31]  10.10 [4.49–15.71] | 1  0.95 [0.38–2.27]  1.10 [0.30–4.11] | 0.870  0.886 |
| Calcifications | no  yes | 219  4 | 11.35 [10.02–12.68]  6.38 [0.00–17.99] | 1  1.15 [0.42–3.10] | 0.789 |
| Necrosis | no  yes | 210  13 | 11.45 [9.38–13.52]  9.11 [6.94–11.29] | 1  1.93 [1.07–3.49] | **0.030** |
| Hyperarteriali-zation | no  yes | 122  101 | 11.55 [8.71–14.38]  10.43 [9.13–11.73] | 1  1.17 [0.87–1.58] | 0.292 |
| Portal vein involvement | no  yes | 204  7 | 12.27 [10.58–13.96]  3.95 [3.61–4.29] | 1  6.11 [2.77–13.51] | **<0.001** |
| Other vessel involvement | no  yes | 199  12 | 12.27 [10.56–13.98]  5.33 [3.43–7.23] | 1  1.86 [1.03–3.35] | **0.040** |
| Ascites | no  yes | 189  21 | 13.36 [11.42–15.29]  6.45 [4.03–8.87] | 1  2.94 [1.83–4.73] | **<0.001** |

*ALT: alanine aminotransferase; AST: aspartate aminotransferase; CRP: C-reactive protein; ECOG: Eastern Cooperative Oncology Group; FDoP: first diagnosis of primary tumor; FDoM: first diagnosis of metastases; GGT: gamma-glutamyl transpeptidase; Hb: hemoglobin; LDH: lactate dehydrogenase; NLR: neutrophil-to-lymphocyte ratio; TLR: thrombocyte-to-lymphocyte ratio; HAIC: hepatic arterial infusion chemotherapy;* *significant values with p<0.05 written in bold*

**Supplemental Table 2**: Results of univariate pre-treatment analysis of time to change treatment strategy (TTCS).

| **Subgroups** |  | **n** | **Median TTCS [95% CI]** | **HR [95% CI]** | **p-value** |
| --- | --- | --- | --- | --- | --- |
| Leukocytes | normal  > 9.2 /nl | 178  55 | 5.99 [5.46–6.52]  3.45 [2.09–4.82] | 1  1.32 [0.96–1.82] | 0.087 |
| Neutrophils | normal  > 6.2 /nl | 168  60 | 6.12 [5.65–6.59]  3.13 [1.15–5.10] | 1  1.46 [1.07–1.99] | **0.017** |
| Lymphocytes | normal  < 1.0 /nl | 192  36 | 5.99 [5.01–6.96]  2.93 [0.00–6.41] | 1  1.57 [1.07–2.31] | **0.022** |
| Thrombocytes | normal  > 380 /nl | 196  37 | 5.99 [5.14–6.83]  3.29 [2.39–4.19] | 1  1.40 [0.97–2.03] | 0.073 |
| Hb | normal  < 12 g/dl | 189  44 | 5.99 [5.45–6.53]  3.16 [2.23–4.08] | 1  1.65 [1.16–2.34] | **0.005** |
| NLR | ≤ median (5)  > median (5) | 182  46 | 6.12 [5.50–6.74]  1.91 [0.79–3.03] | 1  2.38 [1.68–3.36] | **<0.001** |
| TLR | ≤ median (175)  > median (175) | 107  120 | 7.20 [5.37–9.04]  3.55 [2.65–4.46] | 1  1.59 [1.21–2.09] | **<0.001** |
| Bilirubin | normal  > 1.2 mg/dl | 215  17 | 5.76 [4.78–6.73]  4.67 [0.00–10.55] | 1  1.12 [0.68–1.83] | 0.666 |
| AST | ≤ 35 U/l  > 35 U/l | 109  123 | 7.20 [5.60–8.80]  3.55 [2.13–4.98] | 1  1.65 [1.26–2.17] | **<0.001** |
| ALT | ≤ 35 U/l  > 35 U/l | 121  111 | 6.45 [5.28–7.62]  3.68 [2.47–4.90] | 1  1.58 [1.21–2.07] | **<0.001** |
| GGT | ≤ 70 U/l  > 70 U/l | 104  104 | 7.60 [5.63–9.57]  3.29 [2.35–4.23] | 1  1.99 [1.51–2.62] | **<0.001** |
| AP | ≤ 100 U/l  > 100 U/l | 82  83 | 6.71 [5.49–7.93]  3.16 [2.18–4.14] | 1  1.29 [0.99–1.69] | 0.063 |
| LDH | ≤ 400 U/l  > 400 U/l | 123  87 | 7.57 [5.83–9.30]  2.86 [1.56–4.17] | 1  2.37 [1.78–3.16] | **<0.001** |
| Total protein | normal  < 6.4 g/dl | 200  31 | 5.95 [5.21–6.70]  2.86 [0.45–5.28] | 1  1.47 [0.99–2.19] | 0.057 |
| Albumin | normal  < 3.2 g/dl | 150  5 | 6.09 [5.52–6.66]  1.35 [1.00–1.70] | 1  5.57 [2.26–13.72] | **<0.001** |
| CRP | normal  ≥ 0.5 mg/dl | 113  110 | 7.57 [5.82–9.32]  3.52 [2.86–4.18] | 1  1.72 [1.31–2.26] | **<0.001** |
| S-100 | normal  > 0.105 µg/l | 53  51 | 7.60 [5.24–9.96]  3.91 [0.96–6.87] | 1  1.14 [0.84–1.58] | 0.392 |
| ECOG status | 0  1  ≥2 | 151  65  11 | 5.99 [5.46–6.52]  5.89 [3.19–8.58]  1.61 [1.33–1.90] | 1  1.09 [0.81–1.47]  1.79 [0.94–3.40] | 0.586  0.077 |
| Karnofsky Performance Status | 90–100%  70–80%  ≤ 60% | 148  70  8 | 5.99 [5.43–6.55]  5.56 [3.86–7.26]  1.61 [1.34–1.89] | 1  1.50 [1.12–2.00]  1.99 [0.97–4.08] | **0.007**  0.060 |
| Sex | male  female | 113  122 | 5.76 [4.56–6.96]  5.59 [3.97–7.22] | 1  1.14 [0.87–1.49] | 0.357 |
| Affected eye | left  right | 116  117 | 5.79 [4.35–7.23]  5.53 [4.39–6.67] | 1  0.91 [0.69–1.18] | 0.906 |
| Age at FDoP | < 60 years  ≥ 60 years | 115  119 | 4.90 [3.48–6.32]  5.99 [5.03–6.94] | 1  0.99 [0.76–1.30] | 0.955 |
| Age at FDoM | < 63 years  ≥ 63 years | 120  115 | 5.53 [4.03–7.03]  5.89 [4.94–6.83] | 1  0.97 [0.75–1.27] | 0.846 |
| Age at start of HAIC | < 64 years  ≥ 64 years | 122  113 | 5.10 [3.65–6.55]  5.95 [5.26–6.65] | 1  0.95 [0.73–1.24] | 0.705 |
| Time between FDoP & FDoM | < 32 months  ≥ 32 months | 118  116 | 5.82 [4.53–7.12]  5.10 [3.26–6.94] | 1  0.84 [0.64–1.10] | 0.198 |
| Weight | < 75 kg  ≥ 75kg | 88  110 | 6.22 [4.44–8.00]  5.99 [5.29–6.68] | 1  1.07 [0.81–1.41] | 0.626 |
| Metastatic sites | 1  2  3  ≥4 | 96  65  37  37 | 5.79 [3.71–7.87]  4.61 [2.91–6.30]  5.99 [3.16–9.38]  6.28 [3.19–9.38] | 1  0.98 [0.70–1.36]  0.99 [0.67–1.46]  0.83 [0.56–1.27] | 0.889  0.953  0.353 |
| Secondary tumors | 0  ≥1 | 200  35 | 5.59 [4.67–6.52]  6.09 [0.97–11.21 | 1  0.75 [0.50–1.11] | 0.149 |
| Comorbidities | 0  1  2  3  ≥4 | 73  60  56  26  20 | 5.53 [3.25–7.81]  5.89 [4.64–7.14]  5.00 [2.45–7.55]  5.56 [4.23–6.89]  6.71 [0.96–12.47] | 1  0.94 [0.66–1.34]  1.35 [0.94–1.94]  0.95 [0.59–1.55]  0.94 [0.55–1.59] | 0.738  0.100  0.844  0.804 |
| Prior treatments in metastatic UM | 0  1  ≥2 | 159  62  14 | 5.59 [4.42–6.76]  6.09 [5.32–6.85]  1.81 [0.00–4.11] | 1  0.95 [0.70–1.29]  1.63 [0.90–2.95] | 0.735  0.106 |
| Growth pattern | nodular  infiltrative  mixed | 113  28  82 | 3.68 [2.62–4.75]  5.82 [4.99–6.66]  6.12 [4.26–7.98] | 1  1.37 [0.89–2.10]  1.19 [0.89–1.59] | 0.150  0.251 |
| Hepatic nodule size | < 5cm  ≥ 5cm | 143  74 | 6.18 [5.21–7.16]  3.82 [2.28–5.35] | 1  1.55 [1.16–2.06] | **0.003** |
| Density | Isodens  hypodens  hyperdens | 7  202  6 | 1.88 [1.37–2.38]  5.76 [4.77–6.75]  5.59 [0.00–11.63] | 1  0.87 [0.41–1.86]  0.85 [0.27–2.68] | 0.727  0.779 |
| Calcifications | no  yes | 219  4 | 5.76 [4.77–6.74]  3.68 [0.20–7.17] | 1  1.63 [0.61–4.42] | 0.331 |
| Necrosis | no  yes | 210  13 | 5.82 [4.93–6.72]  4.64 [2.44–6.84] | 1  1.52 [0.86–2.67] | 0.147 |
| Hyperarteriali-zation | no  yes | 122  101 | 10.02 [5.68–6.75]  7.79 [2.89–6.46] | 1  1.28 [0.98–1.68] | 0.069 |
| Portal vein involvement | no  yes | 204  7 | 6.12 [5.64–6.60]  1.91 [1.49–2.33] | 1  2.87 [1.33–6.21] | **0.007** |
| Other vessel involvement | no  yes | 199  12 | 6.12 [5.62–6.62]  1.74 [1.41–2.08] | 1  1.47 [0.82–2.64] | 0.197 |
| Ascites | no  yes | 189  21 | 6.18 [5.71–6.66]  2.07 [0.50–3.65] | 1  1.99 [1.26–3.14] | **0.003** |

*ALT: alanine aminotransferase; AST: aspartate aminotransferase; CRP: C-reactive protein; ECOG: Eastern Cooperative Oncology Group; FDoP: first diagnosis of primary tumor; FDoM: first diagnosis of metastases; GGT: gamma-glutamyl transpeptidase; Hb: hemoglobin; LDH: lactate dehydrogenase; NLR: neutrophil-to-lymphocyte ratio; TLR: thrombocyte-to-lymphocyte ratio; HAIC: hepatic arterial infusion chemotherapy; significant values with p<0.05 written in bold*

**Supplemental Table 3**: Results of univariate post first treatment analysis of overall survival (OS).

| **Subgroups** |  | **n** | **Median OS [95% CI]** | **HR [95% CI]** | **p-value** |
| --- | --- | --- | --- | --- | --- |
| Leukocytes | normal  > 9.2 /nl | 193  20 | 12.73 [11.01–14.45]  5.63 [3.84–7.41] | 1  2.01 [1.26–3.51] | **0.005** |
| Neutrophils | normal  > 6.2 /nl | 180  29 | 13.36 [11.36–15.36]  6.28 [4.55–8.02] | 1  2.17 [1.42–3.33] | **<0.001** |
| Lymphocytes | normal  < 1.0 /nl | 108  101 | 13.19 [10.84–15.54]  11.45 [8.93–13.97] | 1  0.98 [0.73–1.32] | 0.884 |
| Thrombocytes | normal  > 380 /nl | 190  23 | 13.16 [11.36–14.96]  5.23 [2.61–7.86] | 1  2.22 [141–3.50] | **<0.001** |
| Hb | normal  < 12 g/dl | 141  72 | 13.98 [11.12–16.84]  9.77 [7.26–12.28] | 1  1.51 [1.09–2.08] | **0.013** |
| NLR | ≤ median (5)  > median (5) | 147  62 | 13.88 [11.62–16.14]  7.07 [5.17–8.98] | 1  1.77 [1.26–2.49] | **<0.001** |
| TLR | ≤ median (175)  > median (175) | 41  168 | 18.52 [12.39–24.65]  11.38 [10.37–12.39] | 1  1.55 [1.04–2.32] | **0.031** |
| Bilirubin | normal  > 1.2 mg/dl | 197  15 | 12.24 [10.40–14.07]  4.70 [0.00–11.39] | 1  1.46 [0.84–2.5 2] | 0.177 |
| AST | ≤ 35 U/l  > 35 U/l | 131  82 | 15.86 [13.32–18.39]  7.17 [6.18–8.17] | 1  2.76 [2.05–3.72] | **<0.001** |
| ALT | ≤ 35 U/l  > 35 U/l | 136  76 | 13.88 [12.10–15.67]  8.85 [6.74–10.96] | 1  1.76 [1.31–2.37] | **<0.001** |
| GGT | ≤ 70 U/l  > 70 U/l | 106  100 | 16.91 [13.71–20.11]  7.80 [6.48–9.11] | 1  2.98 [2.18–4.07] | **<0.001** |
| AP | ≤ 100 U/l  > 100 U/l | 91  65 | 15.82 [13.23–18.41]  7.57 [6.27–8.86] | 1  2.03 [1.49–2.77] | **<0.001** |
| LDH | ≤ 400 U/l  > 400 U/l | 140  89 | 16.84 [14.47–19.21]  6.28 [5.21–7.36] | 1  4.01 [2.94–5.46] | **<0.001** |
| Total protein | normal  < 6.4 g/dl | 180  31 | 12.40 [10.54–14.27]  11.45 [6.77–16.12] | 1  1.26 [0.76–2.09] | 0.369 |
| Albumin | normal  < 3.2 g/dl | 123  2 | 12.24 [9.73–14.74]  2.86 [- – -] | 1  1.57 [0.39–6.36] | 0.525 |
| CRP | normal  ≥ 0.5 mg/dl | 127  77 | 15.82 [13.30–18.35]  8.45 [6.65–10.26] | 1  2.43 [1.80–3.2 7] | **<0.001** |
| S-100 | normal  > 0.105 µg/l | 53  51 | 14.21 [9.49–18.93]  10.30 [7.91–12.68] | 1  4.91 [2.53–9.55] | **<0.001** |
| ECOG status | 0  1  ≥2 | 62  121  43 | 12.24 [8.57–15.91]  11.55 [9.08–14.02]  9.28 [5.00–13.58] | 1  0.90 [0.63–1.29]  1.57 [1.00–2.40] | 0.567  0.039 |
| Karnofsky Performance Status | 90-100%  70-80%  ≤ 60% | 62  121  43 | 12.24 [8.57–15.91]  11.55 [9.08–14.02]  9.28 [5.00–13.58] | 1  0.90 [0.63–1.29]  1.57 [1.00–2.40] | 0.567  0.039 |
| Sex | male  female | 113  122 | 11.02 [8.59–13.45]  11.38 [9.03–13.74] | 1  1.01 [0.75–1.36] | 0.927 |
| Affected eye | left  right | 116  117 | 11.45 [8.67–14.23]  10.46 [9.10–11.82] | 1  0.94 [0.70–1.26] | 0.664 |
| Age at FDoP | < 60 years  ≥ 60 years | 115  119 | 10.53 [9.39–11.66]  12.40 [9.56–15.24] | 1  0.95 [0.70–1.27] | 0.708 |
| Age at FDoM | < 63 years  ≥ 63 years | 120  115 | 11.22 [10.10–12.34]  11.45 [8.46–14.44] | 1  0.97 [0.72–1.30] | 0.835 |
| Age at start of HAIC | < 64 years  ≥ 64 years | 122  113 | 11.18 [10.05–12.32]  12.40 [9.03–15.77] | 1  0.92 [0.69–1.24] | 0.583 |
| Time between FDoP & FDoM | < 32 months  ≥ 32 months | 118  116 | 11.38 [9.65–13.11]  11.45 [9.02–13.88] | 1  0.84 [0.63–1.14] | 0.263 |
| Weight | < 75 kg  ≥ 75kg | 93  110 | 11.22 [9.87–12.57]  12.73 [10.40–15.06] | 1  0.92 [0.68–1.24] | 0.575 |
| Metastatic sites | 1  2  3  ≥4 | 96  65  37  37 | 11.45 [9.25–13.61]  11.18 [7.18–15.19]  10.10 [8.71–11.49]  13.88 [11.35–16.42] | 1  1.10 [0.76–1.60]  1.21 [0.80–1.85]  0.89 [0.57–1.38] | 0.628  0.373  0.595 |
| Secondary tumors | 0  ≥1 | 200  35 | 11.18 [10.22–12.15]  15.82 [9.88–21.77] | 1  0.75 [0.48–1.17] | 0.199 |
| Comorbidities | 0  1  2  3  ≥4 | 73  60  56  26  20 | 13.59 [10.48–16.69]  10.43 [7.30–13.56]  10.26 [9.04–11.49]  11.18 [9.24–13.13]  12.40 [8.60–16.20] | 1  1.13 [0.77–1.65]  1.51 [1.01–2.28]  1.22 [0.72–2.05]  1.21 [0.65–2.26] | 0.545  **0.046**  0.456  0.559 |
| Prior treatments in metastatic UM | 0  1  ≥2 | 159  62  14 | 13.16 [11.21–15.11]  10.16 [8.68–11.65]  8.22 [0.00–16.89] | 1  1.20 [0.86–1.68]  2.67 [1.42–4.99] | 0.273  **0.002** |
| Growth pattern | nodular  infiltrative  mixed | 109  26  76 | 14.24 [12.97–15.51]  10.16 [6.25–14.08]  10.30 [8.61–11.99] | 1  0.57 [0.35–0.92]  0.87 [0.53–1.43] | **0.022**  0.589 |
| Hepatic nodule size | < 5cm  ≥ 5cm | 142  65 | 13.88 [11.71–16.06]  8.85 [6.55–11.15] | 1  1.49 [1.08–2.06] | **0.015** |
| Density | Isodens  hypodens  hyperdens | 13  191  7 | 7.57 [0.00–16.55]  12.24 [10.38–14.09]  10.10 [6.99–13.21] | 1  0.69 [0.36–1.31]  0.92 [0.32–2.70] | 0.251  0.883 |
| Calcifications | no  yes | 207  3 | 11.51 [9.58–13.44]  14.38 [1.58–27.17] | 1  0.87 [0.28–2.74] | 0.815 |
| Necrosis | no  yes | 187  24 | 13.16 [11.32–14.99]  7.80 [5.28–10.31] | 1  2.60 [1.60–4.23] | **<0.001** |
| Hyperarteriali-zation | no  yes | 96  115 | 13.45 [9.60–16.72  11.55 [9.56–13.54] | 1  1.46 [1.07–1.99] | **0.018** |
| Portal vein involvement | no  yes | 204  7 | 12.27 [10.58–13.96]  3.95 [3.61–4.29] | 1  9.31 [4.12–21.05] | **<0.001** |
| Other vessel involvement | no  yes | 199  12 | 12.27 [10.56–13.98]  5.33 [3.43–7.23] | 1  1.86 [1.03–3.35] | **0.040** |
| Ascites | no  yes | 189  21 | 13.36 [11.42–15.29]  6.45 [4.03–8.87] | 1  2.94 [1.83–4.73] | **<0.001** |

*ALT: alanine aminotransferase; AST: aspartate aminotransferase; CRP: C-reactive protein; ECOG: Eastern Cooperative Oncology Group; FDoP: first diagnosis of primary tumor; FDoM: first diagnosis of metastases; GGT: gamma-glutamyl transpeptidase; Hb: hemoglobin; LDH: lactate dehydrogenase; NLR: neutrophil-to-lymphocyte ratio; TLR: thrombocyte-to-lymphocyte ratio; HAIC: hepatic arterial infusion chemotherapy; significant values with p<0.05 written in bold*

**Supplemental Table 4**: Results of multivariate post first treatment analysis of overall survival (OS).

| **Subgroups** |  | **n** | **Median OS [95% CI]** | **HR [95% CI]** | **p-value** |
| --- | --- | --- | --- | --- | --- |
| Hb | normal  < 12 g/dl | 141  72 | 13.98 [11.12–16.84]  9.77 [7.26–12.28] | 1  0.94 [0.61–1.44] | 0.773 |
| NLR | ≤ median (5)  > median (5) | 147  62 | 13.88 [11.62–16.14]  7.07 [5.17–8.98] | 1  1.09 [0.72–1.67] | 0.686 |
| TLR | ≤ median (175)  > median (175) | 41  168 | 18.52 [12.39–24.65]  11.38 [10.37–12.39] | 1  1.09 [0.69–1.72] | 0.720 |
| AST | ≤ 35 U/l  > 35 U/l | 131  82 | 15.86 [13.32–18.39]  7.17 [6.18–8.17] | 1  1.07 [0.66–1.74] | 0.779 |
| ALT | ≤ 35 U/l  > 35 U/l | 136  76 | 13.88 [12.10–15.67]  8.85 [6.74–10.96] | 1  1.05 [0.70–1.56] | 0.828 |
| GGT | ≤ 70 U/l  > 70 U/l | 106  100 | 16.91 [13.71–20.11]  7.80 [6.48–9.11] | 1  1.57 [1.01–2.43] | **0.043** |
| AP | ≤ 100 U/l  > 100 U/l | 91  65 | 15.82 [13.23–18.41]  7.57 [6.27–8.86] | 1  1.27 [0.82–1.97] | 0.278 |
| LDH | ≤ 400 U/l  > 400 U/l | 140  89 | 16.84 [14.47–19.21]  6.28 [5.21–7.36] | 1  2.67 [1.76–4.07] | **<0.001** |
| CRP | normal  ≥ 0.5 mg/dl | 127  77 | 15.82 [13.30–18.35]  8.45 [6.65–10.26] | 1  1.72 [1.19–2.49] | **0.004** |
| Karnofsky Performance Status | 90–100%  70–80%  ≤ 60% | 62  121  43 | 12.24 [8.57–15.91]  11.55 [9.08–14.02]  9.28 [5.00–13.58] | 1  0.89 [0.59–1.34]  1.54 [0.94–2.54] | 0.567  0.088 |
| Prior treatments in metastatic UM | 0  1  ≥2 | 159  62  14 | 13.16 [11.21–15.11]  10.16 [8.68–11.65]  8.22 [0.00–16.89] | 1  1.27 [0.87–1.85]  1.68 [0.82–3.42] | 0.219  0.154 |
| Growth pattern | nodular  infiltrative  mixed | 109  26  76 | 14.24 [12.97–15.51]  10.16 [6.25–14.08]  10.30 [8.61–11.99] | 1  0.98 [0.55–1.77]  1.40 [0.80–2.42] | 0.954  0.236 |
| Hepatic nodule size | < 5cm  ≥ 5cm | 142  65 | 13.88 [11.71–16.06]  8.85 [6.55–11.15] | 1  1.23 [0.81–1.85] | 0.332 |
| Necrosis | no  yes | 187  24 | 13.16 [11.32–14.99]  7.80 [5.28–10.31] | 1  1.21 [0.70–2.10] | 0.504 |
| Hyperarterialization | no  yes | 96  115 | 13.45 [9.60–16.72  11.55 [9.56–13.54] | 1  1.20 [0.82–1.75] | 0.350 |
| Portal vein involvement | no  yes | 204  7 | 12.27 [10.58–13.96]  3.95 [3.61–4.29] | 1  1.37 [0.47–3.99] | 0.567 |
| Other vessel involvement | no  yes | 199  12 | 12.27 [10.56–13.98]  5.33 [3.43–7.23] | 1  0.93 [0.45–1.95] | 0.853 |
| Ascites | no  yes | 189  21 | 13.36 [11.42–15.29]  6.45 [4.03–8.87] | 1  1.58 [0.86–2.89] | 0.140 |

*ALT: alanine aminotransferase; AST: aspartate aminotransferase; CRP: C-reactive protein; ECOG: Eastern Cooperative Oncology Group; FDoP: first diagnosis of primary tumor; FDoM: first diagnosis of metastases; GGT: gamma-glutamyl transpeptidase; Hb: hemoglobin; LDH: lactate dehydrogenase; NLR: neutrophil-to-lymphocyte ratio; TLR: thrombocyte-to-lymphocyte ratio; HAIC: hepatic arterial infusion chemotherapy; significant values with p<0.05 written in bold*

**Supplemental Table 5**: Results of univariate post first treatment analysis of time to change treatment strategy (TTCS).

| **Subgroups** |  | **n** | **Median TTCS [95% CI]** | **HR [95% CI]** | **p-value** |
| --- | --- | --- | --- | --- | --- |
| Leukocytes | normal  > 9.2 /nl | 193  20 | 6.18 [5.68–6.69]  3.60 [2.02–4.10] | 1  1.39 [0.86–2.26] | 0.182 |
| Neutrophils | normal  > 6.2 /nl | 180  29 | 6.45 [5.59–7.30]  2.93 [1.36–4.50] | 1  2.05 [1.36–3.11] | **<0.001** |
| Lymphocytes | normal  < 1.0 /nl | 101  108 | 6.48 [5.26–7.70]  5.76 [4.50–7.01] | 1  0.99 [0.76–1.30] | 0.939 |
| Thrombocytes | normal  > 380 /nl | 190  23 | 6.28 [5.61–6.96]  1.91 [0.16–3.66] | 1  1.36 [0.87–2.12] | 0.174 |
| Hb | normal  < 12 g/dl | 141  72 | 6.71 [5.55–7.88]  3.68 [2.21–5.16] | 1  1.53 [1.14–2.07] | **0.005** |
| NLR | ≤ median (5)  > median (5) | 147  62 | 7.37 [6.21–8.52]  3.13 [2.30–3.95] | 1  1.90 [1.38–2.60] | **<0.001** |
| TLR | ≤ median (175)  > median (175) | 41  168 | 9.31 [4.73–13.90]  5.88 [4.99–6.79] | 1  1.48 [1.04–2.11] | **0.029** |
| Bilirubin | normal  > 1.2 mg/dl | 197  15 | 6.09 [5.52–6.65]  1.68 [1.39–1.97] | 1  1.62 [0.96–2.75] | 0.073 |
| AST | ≤ 35 U/l  > 35 U/l | 131  82 | 7.37 [5.79–8.95]  3.55 [2.27–4.84] | 1  2.25 [1.71–2.96] | **<0.001** |
| ALT | ≤ 35 U/l  > 35 U/l | 136  76 | 6.55 [5.40–7.69]  4.67 [2.72–6.63] | 1  1.72 [1.31–2.25] | **<0.001** |
| GGT | ≤ 70 U/l  > 70 U/l | 106  100 | 9.01 [7.36–10.67]  3.49 [2.43–4.54] | 1  2.50 [1.90–3.30] | **<0.001** |
| AP | ≤ 100 U/l  > 100 U/l | 89  67 | 7.37 [4.79–9.95]  3.45 [2.23–4.68] | 1  1.75 [1.29–2.36] | **<0.001** |
| LDH | ≤ 400 U/l  > 400 U/l | 140  89 | 7.83 [6.13–9.53]  1.88 [0.94–2.81] | 1  3.18 [2.39–4.23] | **<0.001** |
| Total protein | normal  < 6.4 g/dl | 180  31 | 6.18 [5.55–6.82]  4.38 [2.20–6.55] | 1  1.28 [0.85–1.92] | 0.232 |
| Albumin | normal  < 3.2 g/dl | 123  2 | 6.12 [5.52–6.72]  1.68 [-–-] | 1  1.12 [0.28–4.53] | 0.871 |
| CRP | normal  ≥ 0.5 mg/dl | 127  77 | 7.83 [6.14–9.52]  3.29 [2.71–3.87] | 1  2.09 [1.60–2.74] | **<0.001** |
| S-100 | normal  > 0.105 µg/l | 53  51 | 7.60 [5.24–9.96]  3.91 [0.96–6.87] | 1  3.22 [1.69–6.16] | **<0.001** |
| ECOG status | 0  1  ≥2 | 62  121  43 | 6.09 [3.45–8.72]  5.76 [4.51–7.00]  5.10 [2.90–7.30] | 1  0.87 [0.63–1.19]  1.14 [0.76–1.71 | 0.381  0.519 |
| Karnofsky Performance Status | 90–100%  70–80%  ≤ 60% | 62  121  43 | 6.09 [3.45–8.72]  5.76 [4.51–7.00]  5.10 [2.90–7.30] | 1  0.87 [0.63–1.19]  1.14 [0.76–1.71] | 0.381  0.519 |
| Sex | male  female | 113  122 | 5.76 [4.56–6.96]  5.59 [3.97–7.22] | 1  1.14 [0.87–1.49] | 0.357 |
| Affected eye | left  right | 116  117 | 5.79 [4.35–7.23]  5.53 [4.39–6.67] | 1  0.91 [0.69–1.18] | 0.906 |
| Age at FDoP | < 60 years  ≥ 60 years | 115  119 | 4.90 [3.48–6.32]  5.99 [5.03–6.94] | 1  0.99 [0.76–1.30] | 0.955 |
| Age at FDoM | < 63 years  ≥ 63 years | 120  115 | 5.53 [4.03–7.03]  5.89 [4.94–6.83] | 1  0.97 [0.75–1.27] | 0.846 |
| Age at start of HAIC | < 64 years  ≥ 64 years | 122  113 | 5.10 [3.65–6.55]  5.95 [5.26–6.65] | 1  0.95 [0.73–1.24] | 0.705 |
| Time between FDoP & FDoM | < 32 months  ≥ 32 months | 118  116 | 5.82 [4.53–7.12]  5.10 [3.26–6.94] | 1  0.84 [0.64–1.10] | 0.198 |
| Weight | < 75 kg  ≥ 75kg | 93  110 | 5.99 [4.14–7.84]  5.99 [5.29–6.69] | 1  0.94 [0.72–1.24] | 0.666 |
| Metastatic sites | 1  2  3  ≥4 | 96  65  37  37 | 5.79 [3.71–7.87]  4.61 [2.91–6.30]  5.99 [3.16–9.38]  6.28 [3.19–9.38] | 1  0.98 [0.70–1.36]  0.99 [0.67–1.46]  0.83 [0.56–1.27] | 0.889  0.953  0.353 |
| Secondary tumors | 0  ≥1 | 200  35 | 5.59 [4.67–6.52]  6.09 [0.97–11.21 | 1  0.75 [0.50–1.11] | 0.149 |
| Comorbidities | 0  1  2  3  ≥4 | 73  60  56  26  20 | 5.53 [3.25–7.81]  5.89 [4.64–7.14]  5.00 [2.45–7.55]  5.56 [4.23–6.89]  6.71 [0.96–12.47] | 1  0.94 [0.66–1.34]  1.35 [0.94–1.94]  0.95 [0.59–1.55]  0.94 [0.55–1.59] | 0.738  0.100  0.844  0.804 |
| Prior treatments in metastatic UM | 0  1  ≥2 | 159  62  14 | 5.59 [4.42–6.76]  6.09 [5.32–6.85]  1.81 [0.00–4.11] | 1  0.95 [0.70–1.29]  1.63 [0.90–2.95] | 0.735  0.106 |
| Growth pattern | nodular  infiltrative  mixed | 109  26  76 | 6.09 [5.45–6.73]  5.10 [3.28–6.92]  5.99 [4.31–7.66] | 1  0.97 [0.63–1.51]  1.00 [0.63–1.60] | 0.896  0.899 |
| Hepatic nodule size | < 5cm  ≥ 5cm | 142  65 | 6.45 [5.34–7.56]  3.68 [2.39–4.98] | 1  1.39 [1.03–1.87] | **0.031** |
| Density | Isodens  hypodens  hyperdens | 13  191  7 | 3.13 [0.95–5.30]  6.09 [5.53–6.64]  5.76 [5.50–6.01] | 1  0.69 [0.38–1.24]  0.72 [0.27–1.92] | 0.215  0.509 |
| Calcifications | no  yes | 207  3 | 5.99 [5.48–6.50]  5.10 [2.84–7.36] | 1  1.31 [0.48–4.12] | 0.641 |
| Necrosis | no  yes | 187  24 | 6.22 [5.68–6.76]  3.22 [2.04–4.40] | 1  1.87 [1.20–2.91] | **0.005** |
| Hyperarteriali-zation | no  yes | 96  115 | 6.12 [5.66–6.57]  5.76 [4.47–7.04] | 1  1.29 [0.98–1.69] | 0.070 |
| Portal vein involvement | no  yes | 204  7 | 6.12 [5.64–6.60]  1.91 [1.49–2.33] | 1  2.87 [1.33–6.21] | **0.007** |
| Other vessel involvement | no  yes | 199  12 | 6.12 [5.62–6.62]  1.74 [1.41–2.08] | 1  1.47 [0.82–2.64] | 0.197 |
| Ascites | no  yes | 189  21 | 6.18 [5.71–6.66]  2.07 [0.50–3.65] | 1  1.99 [1.26–3.14] | **0.003** |

*ALT: alanine aminotransferase; AST: aspartate aminotransferase; CRP: C-reactive protein; ECOG: Eastern Cooperative Oncology Group; FDoP: first diagnosis of primary tumor; FDoM: first diagnosis of metastases; GGT: gamma-glutamyl transpeptidase; Hb: hemoglobin; LDH: lactate dehydrogenase; NLR: neutrophil-to-lymphocyte ratio; TLR: thrombocyte-to-lymphocyte ratio; HAIC: hepatic arterial infusion chemotherapy; significant values with p<0.05 written in bold*

**Supplemental Table 6**: Results of multivariate post first treatment analysis of time to change treatment strategy (TTCS).

| **Subgroups** |  | **n** | **Median TTCS [95% CI]** | **HR [95% CI]** | **p-value** |
| --- | --- | --- | --- | --- | --- |
| Hb | normal  < 12 g/dl | 141  72 | 6.71 [5.55–7.88]  3.68 [2.21–5.16] | 1  1.29 [0.89–1.88] | 0.173 |
| NLR | ≤ median (5)  > median (5) | 147  62 | 7.37 [6.21–8.52]  3.13 [2.30–3.95] | 1  1.55 [1.06–2.19] | **0.023** |
| TLR | ≤ median (175)  > median (175) | 41  168 | 9.31 [4.73–13.90]  5.88 [4.99–6.79] | 1  1.15 [0.78–1.71] | 0.472 |
| AST | ≤ 35 U/l  > 35 U/l | 131  82 | 7.37 [5.79–8.95]  3.55 [2.27–4.84] | 1  0.89 [0.58–1.36] | 0.586 |
| ALT | ≤ 35 U/l  > 35 U/l | 136  76 | 6.55 [5.40–7.69]  4.67 [2.72–6.63] | 1  1.34 [0.95–1.90] | 0.091 |
| GGT | ≤ 70 U/l  > 70 U/l | 106  100 | 9.01 [7.36–10.67]  3.49 [2.43–4.54] | 1  1.72 [1.24–2.39] | **0.001** |
| AP | ≤ 100 U/l  > 100 U/l | 89  67 | 7.37 [4.79–9.95]  3.45 [2.23–4.68] | 1  0.84 [0.59–1.21] | 0.349 |
| LDH | ≤ 400 U/l  > 400 U/l | 140  89 | 7.83 [6.13–9.53]  1.88 [0.94–2.81] | 1  2.23 [1.51–3.31] | **<0.001** |
| CRP | normal  ≥ 0.5 mg/dl | 127  77 | 7.83 [6.14–9.52]  3.29 [2.71–3.87] | 1  1.62 [1.18–2.21] | **0.003** |
| Hepatic nodule size | < 5cm  ≥ 5cm | 142  65 | 6.45 [5.34–7.56]  3.68 [2.39–4.98] | 1  1.19 [0.84–1.68] | 0.319 |
| Necrosis | no  yes | 187  24 | 6.22 [5.68–6.76]  3.22 [2.04–4.40] | 1  1.37 [0.85–2.18] | 0.193 |
| Portal vein involvement | no  yes | 204  7 | 6.12 [5.64–6.60]  1.91 [1.49–2.33] | 1  0.66 [0.26–1.53] | 0.312 |
| Ascites | no  yes | 189  21 | 6.18 [5.71–6.66]  2.07 [0.50–3.65] | 1  1.42 [0.72–2.14] | 0.435 |

*ALT: alanine aminotransferase; AST: aspartate aminotransferase; CRP: C-reactive protein; ECOG: Eastern Cooperative Oncology Group; FDoP: first diagnosis of primary tumor; FDoM: first diagnosis of metastases; GGT: gamma-glutamyl transpeptidase; Hb: hemoglobin; LDH: lactate dehydrogenase; NLR: neutrophil-to-lymphocyte ratio; TLR: thrombocyte-to-lymphocyte ratio; HAIC: hepatic arterial infusion chemotherapy; significant values with p<0.05 written in bold*

**Supplemental Table 7:** Performance of ML-based models for prediction of overall survival (OS).

| **Data used** | **Sensitivity** | **Specificity** | **Precision** | **Accuracy** |
| --- | --- | --- | --- | --- |
| **PRE data** |  |  |  |  |
| Clinical | 0.58 ± 0.07 | 0.65 ± 0.16 | 0.64 ± 0.12 | 0.60 ± 0.05 |
| Pyradiomics | 0.59 ± 0.21 | 0.60 ± 0.19 | 0.59 ± 0.14 | 0.57 ± 0.06 |
| BN features | 0.46 ± 0.19 | 0.61 ± 0.12 | 0.51 ± 0.10 | 0.53 ± 0.10 |
| Clinical+Pyradiomics | 0.58 ± 0.13 | 0.64 ± 0.15 | 0.61 ± 0.14 | 0.61 ± 0.06 |
| Clinical+BN features | 0.58 ± 0.21 | 0.64 ± 0.15 | 0.61 ± 0.11 | 0.59 ± 0.12 |
| Clinical+Noise | 0.47 ± 0.19 | 0.50 ± 0.27 | 0.52 ± 0.20 | 0.45 ± 0.06 |
| **POST data** |  |  |  |  |
| Clinical | 0.63 ± 0.15 | 0.61 ± 0.14 | 0.63 ± 0.11 | 0.61 ± 0.07 |
| Pyradiomics | 0.60 ± 0.13 | 0.64 ± 0.10 | 0.63 ± 0.16 | 0.60 ± 0.05 |
| BN features | 0.58 ± 0.15 | 0.42 ± 0.18 | 0.52 ± 0.14 | 0.49 ± 0.11 |
| Clinical+Pyradiomics | 0.69 ± 0.10 | 0.60 ± 0.14 | 0.65 ± 0.08 | 0.65 ± 0.07 |
| Clinical+BN features | 0.64 ± 0.14 | 0.55 ± 0.19 | 0.61 ± 0.11 | 0.59 ± 0.10 |
| Clinical+Noise | 0.56 ± 0.21 | 0.50 ± 0.26 | 0.55 ± 0.26 | 0.51 ± 0.18 |
| **PRE+POST data** |  |  |  |  |
| Clinical | 0.59 ± 0.23 | 0.70 ± 0.08 | 0.66 ± 0.13 | 0.64 ± 0.09 |
| Pyradiomics | 0.57 ± 0.19 | 0.68 ± 0.20 | 0.69 ± 0.21 | 0.62 ± 0.10 |
| BN features | 0.56 ± 0.18 | 0.52 ± 0.13 | 0.53 ± 0.06 | 0.53 ± 0.07 |
| Clinical+Pyradiomics | 0.65 ± 0.23 | 0.66 ± 0.19 | 0.68 ± 0.10 | 0.64 ± 0.11 |
| Clinical+BN features | 0.64 ± 0.21 | 0.66 ± 0.15 | 0.66 ± 0.13 | 0.64 ± 0.11 |
| Clinical+Noise | 0.71 ± 0.21 | 0.48 ± 0.16 | 0.59 ± 0.10 | 0.59 ± 0.10 |

**Supplemental Table 8**: Accuracy of the ML-based models for prediction of time to change treatment strategy (TTCS) in the four different classes.

|  | **Class** | | | |
| --- | --- | --- | --- | --- |
| **Data used** | **0-3 months** | **3-6 months** | **6-12 months** | **>12 months** |
| **PRE data only** |  |  |  |  |
| Clinical | 0.64 ± 0.03 | 0.70 ± 0.03 | 0.60 ± 0.01 | 0.73 ± 0.03 |
| Pyradiomics | 0.75 ± 0.02 | 0.57 ± 0.03 | 0.56 ± 0.05 | 0.85 ± 0.03 |
| BN features | 0.42 ± 0.07 | 0.80 ± 0.06 | 0.63 ± 0.06 | 0.75 ± 0.03 |
| Clinical+Pyradiomics | 0.73 ± 0.05 | 0.60 ± 0.04 | 0.56 ± 0.05 | 0.84 ± 0.03 |
| Clinical+BN features | 0.52 ± 0.12 | 0.80 ± 0.04 | 0.64 ± 0.06 | 0.76 ± 0.02 |
| Clinical+Noise | 0.48 ± 0.07 | 0.76 ± 0.03 | 0.61 ± 0.03 | 0.81 ± 0.03 |
| **POST data only** |  |  |  |  |
| Clinical | 0.75 ± 0.03 | 0.74 ± 0.03 | 0.63 ± 0.02 | 0.77 ± 0.01 |
| Pyradiomics | 0.73 ± 0.04 | 0.61 ± 0.04 | 0.59 ± 0.03 | 0.82 ± 0.02 |
| BN features | 0.73 ± 0.05 | 0.71 ± 0.04 | 0.40 ± 0.10 | 0.80 ± 0.03 |
| Clinical+Pyradiomics | 0.75 ± 0.02 | 0.68 ± 0.04 | 0.62 ± 0.04 | 0.84 ± 0.02 |
| Clinical+BN features | 0.70 ± 0.05 | 0.76 ± 0.04 | 0.47 ± 0.06 | 0.77 ± 0.02 |
| Clinical+Noise | 0.52 ± 0.02 | 0.79 ± 0.01 | 0.63 ± 0.03 | 0.80 ± 0.01 |
| **PRE+POST data** |  |  |  |  |
| Clinical | 0.75 ± 0.02 | 0.76 ± 0.02 | 0.62 ± 0.02 | 0.73 ± 0.03 |
| Pyradiomics | 0.74 ± 0.01 | 0.57 ± 0.03 | 0.57 ± 0.07 | 0.86 ± 0.02 |
| BN features | 0.80 ± 0.04 | 0.81 ± 0.05 | 0.45 ± 0.09 | 0.78 ± 0.07 |
| Clinical+Pyradiomics | 0.77 ± 0.05 | 0.65 ± 0.03 | 0.55 ± 0.05 | 0.83 ± 0.02 |
| Clinical+BN features | 0.75 ± 0.08 | 0.75 ± 0.05 | 0.44 ± 0.05 | 0.83 ± 0.07 |
| Clinical+Noise | 0.52 ± 0.05 | 0.74 ± 0.03 | 0.62 ± 0.03 | 0.80 ± 0.03 |

**Supplemental Table 9**: Global performance (micro-averages) of the ML-based models for prediction of time to change treatment strategy (TTCS).

| **Data used** | **Sensitivity** | **Specificity** | **Precision** | **Accuracy** | **Overall accuracy** |
| --- | --- | --- | --- | --- | --- |
| **PRE data only** |  |  |  |  |  |
| Clinical | 0.33 ± 0.02 | 0.78 ± 0.01 | 0.33 ± 0.02 | 0.67 ± 0.01 | 0.33 ± 0.02 |
| Pyradiomics | 0.37 ± 0.05 | 0.79 ± 0.02 | 0.37 ± 0.05 | 0.68 ± 0.03 | 0.37 ± 0.05 |
| BN features | 0.30 ± 0.06 | 0.77 ± 0.02 | 0.30 ± 0.06 | 0.65 ± 0.03 | 0.30 ± 0.06 |
| Clinical+Pyradiomics | 0.36 ± 0.06 | 0.79 ± 0.02 | 0.36 ± 0.06 | 0.68 ± 0.03 | 0.36 ± 0.06 |
| Clinical+BN features | 0.36 ± 0.10 | 0.79 ± 0.03 | 0.36 ± 0.10 | 0.68 ± 0.05 | 0.36 ± 0.10 |
| Clinical+Noise | 0.33 ± 0.04 | 0.77 ± 0.01 | 0.33 ± 0.04 | 0.66 ± 0.02 | 0.33 ± 0.04 |
| **POST data only** |  |  |  |  |  |
| Clinical | 0.45 ± 0.04 | 0.81 ± 0.01 | 0.45 ± 0.04 | 0.72 ± 0.02 | 0.45 ± 0.04 |
| Pyradiomics | 0.37 ± 0.04 | 0.79 ± 0.01 | 0.37 ± 0.04 | 0.69 ± 0.02 | 0.37 ± 0.04 |
| BN features | 0.32 ± 0.05 | 0.77 ± 0.01 | 0.32 ± 0.05 | 0.66 ± 0.02 | 0.32 ± 0.05 |
| Clinical+Pyradiomics | 0.44 ± 0.04 | 0.81 ± 0.01 | 0.44 ± 0.04 | 0.72 ± 0.02 | 0.44 ± 0.04 |
| Clinical+BN features | 0.35 ± 0.02 | 0.78 ± 0.01 | 0.35 ± 0.02 | 0.67 ± 0.01 | 0.35 ± 0.02 |
| Clinical+Noise | 0.37 ± 0.03 | 0.79 ± 0.01 | 0.37 ± 0.03 | 0.68 ± 0.01 | 0.37 ± 0.03 |
| **PRE+POST data** |  |  |  |  |  |
| Clinical | 0.43 ± 0.03 | 0.81 ± 0.01 | 0.43 ± 0.03 | 0.71 ± 0.01 | 0.43 ± 0.03 |
| Pyradiomics | 0.37 ± 0.04 | 0.79 ± 0.01 | 0.37 ± 0.04 | 0.68 ± 0.02 | 0.37 ± 0.04 |
| BN features | 0.42 ± 0.07 | 0.81 ± 0.02 | 0.42 ± 0.07 | 0.71 ± 0.03 | 0.42 ± 0.07 |
| Clinical+Pyradiomics | 0.40 ± 0.04 | 0.80 ± 0.01 | 0.40 ± 0.04 | 0.70 ± 0.02 | 0.40 ± 0.04 |
| Clinical+BN features | 0.39 ± 0.08 | 0.79 ± 0.03 | 0.39 ± 0.08 | 0.69 ± 0.04 | 0.39 ± 0.08 |
| Clinical+Noise | 0.34 ± 0.05 | 0.78 ± 0.02 | 0.34 ± 0.05 | 0.67 ± 0.02 | 0.34 ± 0.05 |

**3. Supplemental Figures**

**Supplemental Figure 1**	*Kaplan-Meier analysis for overall survival (OS) in relation to independent prognostic variables identified in post first treatment multivariate analysis.*

*Shown are Kaplan-Meier curves for OS in patients stratified based on (****A****) post first treatment lactate dehydrogenase (LDH) levels, (****B****) post first treatment c-reactive protein (CRP) levels, (****C****) post first treatment gamma-glutamyl transpeptidase (GGT) levels, and (****D****) presence of one or more of the unfavorable prognostic factors shown in A-C. The indicated p-values were determined by comparison of the different groups using the log-rank test.*

**Supplemental Figure 2**	*Kaplan-Meier analysis for time to change treatment strategy (TTCS) in relation to independent prognostic variables identified in post first treatment multivariate analysis.*

*Shown are Kaplan-Meier curves for TTCS in patients stratified based on (****A****) post first treatment lactate dehydrogenase (LDH) levels, (****B****) post first treatment gamma-glutamyl transpeptidase (GGT) levels, (****C****) post first treatment c-reactive protein (CRP) levels, (****D****) post first treatment neutrophil-to-lymphocyte ratio (NLR), and (****E****) presence of one or more of the unfavorable prognostic factors shown in A-D. The indicated p-values were determined by comparison of the different groups using the log-rank test.*

**4. References**

1.	van Griethuysen, J.J.M.; Fedorov, A.; Parmar, C.; Hosny, A.; Aucoin, N.; Narayan, V.; Beets-Tan, R.G.H.; Fillion-Robin, J.-C.; Pieper, S.; Aerts, H.J.W.L. Computational Radiomics System to Decode the Radiographic Phenotype. *Cancer Res.* **2017**, *77*, e104–e107, doi:10.1158/0008-5472.CAN-17-0339.

2.	Pedregosa, F.; Varoquaux, G.; Gramfort, A.; Michel, V.; Thirion, B.; Grisel, O.; Blondel, M.; Prettenhofer, P.; Weiss, R.; Dubourg, V.; et al. Scikit-Learn: Machine Learning in Python. *J. Mach. Learn. Res.* **2011**, *12*, 2825–2830.

3.	Khoja, L.; Atenafu, E.G.; Suciu, S.; Leyvraz, S.; Sato, T.; Marshall, E.; Keilholz, U.; Zimmer, L.; Patel, S.P.; Piperno-Neumann, S.; et al. Meta-Analysis in Metastatic Uveal Melanoma to Determine Progression Free and Overall Survival Benchmarks: An International Rare Cancers Initiative (IRCI) Ocular Melanoma Study. *Ann. Oncol.* **2019**, *30*, 1370–1380, doi:10.1093/annonc/mdz176.

4.	Eskelin, S.; Pyrhönen, S.; Hahka-Kemppinen, M.; Tuomaala, S.; Kivelä, T. A Prognostic Model and Staging for Metastatic Uveal Melanoma. *Cancer* **2003**, *97*, 465–475, doi:10.1002/cncr.11113.

5.	Valpione, S.; Moser, J.C.; Parrozzani, R.; Bazzi, M.; Mansfield, A.S.; Mocellin, S.; Pigozzo, J.; Midena, E.; Markovic, S.N.; Aliberti, C.; et al. Development and External Validation of a Prognostic Nomogram for Metastatic Uveal Melanoma. *PLoS One* **2015**, *10*, e0120181, doi:10.1371/journal.pone.0120181.

6.	Kivelä, T.T.; Piperno-Neumann, S.; Desjardins, L.; Schmittel, A.; Bechrakis, N.; Midena, E.; Leyvraz, S.; Zografos, L.; Grange, J.-D.; Ract-Madoux, G.; et al. Validation of a Prognostic Staging for Metastatic Uveal Melanoma: A Collaborative Study of the European Ophthalmic Oncology Group. *Am. J. Ophthalmol.* **2016**, *168*, 217–226, doi:10.1016/j.ajo.2016.06.002.
